# Engineered extracellular vesicle-encapsulated CHIP as novel nanotherapeutics for treatment of renal fibrosis

**DOI:** 10.1038/s41536-024-00348-0

**Published:** 2024-01-13

**Authors:** Cheng Ji, Jiahui Zhang, Linru Shi, Hui Shi, Wenrong Xu, Jianhua Jin, Hui Qian

**Affiliations:** 1 Wujin Institute of Molecular Diagnostics and Precision Cancer Medicine of Jiangsu University, Wujin Hospital Affiliated with Jiangsu University, Chang Zhou, Jiangsu China; 2 Jiangsu Key Laboratory of Medical Science and Laboratory Medicine, School of Medicine, Department of laboratory Medicine, Jiangsu University, Zhenjiang, China

**Keywords:** Renal fibrosis, Regeneration, Mesenchymal stem cells

## Abstract

Renal interstitial fibrosis (RIF) is a fundamental pathological feature of chronic kidney disease (CKD). However, toxicity and poor renal enrichment of fibrosis inhibitors limit their further applications. In this study, a platform for CKD therapy is developed using superparamagnetic iron oxide nanoparticles (SPION) decorated mesenchymal stem cells derived extracellular vesicles with carboxyl terminus of Hsc70-interacting protein (CHIP) high expression (SPION-EVs) to achieve higher renal-targeting antifibrotic therapeutic effect. SPION-EVs selectively accumulate at the injury renal sites under an external magnetic field. Moreover, SPION-EVs deliver CHIP to induce Smad2/3 degradation in renal tubular cells which alleviates Smad2/3 activation-mediated fibrosis-like changes and collagen deposition. The extracellular vesicle engineering technology provides a potential nanoplatform for RIF therapy through CHIP-mediated Smad2/3 degradation.

## Introduction

With the continuous improvement of the global economy, the prevalence of chronic kidney disease (CKD) continues to elevate which has emerged as a severe public health problem, ≈15% of people suffer from CKD^1,2^. CKD is typically characterized by renal interstitial fibrosis and excessive extracellular matrix deposition contributing to the progressive loss of renal function^3^. Unfortunately, existing effective clinical treatments only alleviate the process of renal fibrosis but do not complete the cure for the disease^4^. Therefore, developing a method for anti-renal fibrosis has very important clinical significance^5^.

Numerous studies have reported that Smad2/3 positively regulates cell fibrosis-like change and inflammation in renal interstitial fibrosis^6,7^. In addition, expression of Smad2/3 in the nucleus can aggregates in the renal tubular cell and accelerate CKD progression^8,9^. Therefore, the key to anti-fibrosis is to regulate the expression of Smad2/3 protein. A recent study revealed that carboxyl terminus of Hsp70 interacting protein (CHIP) as an E3 ubiquitin ligase, may directly participate in regulating the stability of Smad2/3 protein and inhibiting tumorigenesis^10,11^. This finding suggests that increasing the activity of CHIP in the renal tubular cells to promote the clearance of Smad2/3 may be important for RIF therapy. However, CHIP ligase was susceptible to biochemical and physical instability, which may induce to inhibit Smad2/3 activity weakness and lead to decrease the therapeutic efficacy^12^. Moreover, the low enrichment in the renal for CHIP-mediated ubiquitin degradation significantly limits their research and the future clinical transformation applications for RIF therapy^13^. The key to solving problems is to improve CHIP activity and selectively rich in the injured kidney tissue to promote Smad2/3 ubiquitination and degradation.

Extracellular vesicles (EVs) such as microvesicles, are double membrane microparticles (60–1000 nm in size) secreted by cells in a constitutive or inducible manner^14,15^. The released EVs naturally function as intercellular messengers by selecting transporting nucleic acids and proteins to distal or nearby recipient cells^16^. A large number of studies have shown the potential of using EVs as mighty and feasible nanocarriers for drug delivery vector in various situations, from tumor therapy to gene regeneration therapy^17,18^. Compared with current delivery systems, EVs have a unique advantage in the natural origin, which enables them to escape phagocytosis, extend the half-life of therapeutic agents, and low immunogenicity^19^. In addition, magnetic targeted therapy has become a novel research hotspot in recent years, and magnetic nanoparticles have small size and magnetic guided properties^20^, and targeted migration of tissue damage sites under the action of magnetic fields has broad application prospects^21^. Engineered EVs have shown great potential as a promising therapeutic platform and have attracted considerable attention in tissue regeneration^22^. EVs could be engineered to overexpress various proteins in targeting tissues and active signals pathway for the regulation of fibrosis like cells^23^. Therefore, we use superparamagnetic iron oxide nanoparticles to modify engineering EVs loaded CHIP delivery system to enhance anti-fibrosis therapeutic effect for the effectively treatment of CKD.

Here, we provide the valid method for SPION decorated CHIP high-expressing MSC-EVs in CKD renal fibrosis treatment. We found that SPION-EVs-CHIP showed great ability to target injury renal in unilateral ureteral obstruction (UUO) rat. More importantly, SPION-EVs-CHIP significantly reversed collagen deposition by inducing Smad2/3 ubiquitination and degradation of renal tubular cells and inhibiting tubular damage-mediated inflammatory responses as compared to MSC-EVs. Our EV-engineering technology demonstrated that SPION-EVs with CHIP overexpression provide a potential platform for effective renal interstitial fibrosis therapy by inducing Smad2/3 degradation.

## Results

### Characteristics of MSC derived extracellular vesicles with CHIP high expression

**Fig. 1:**
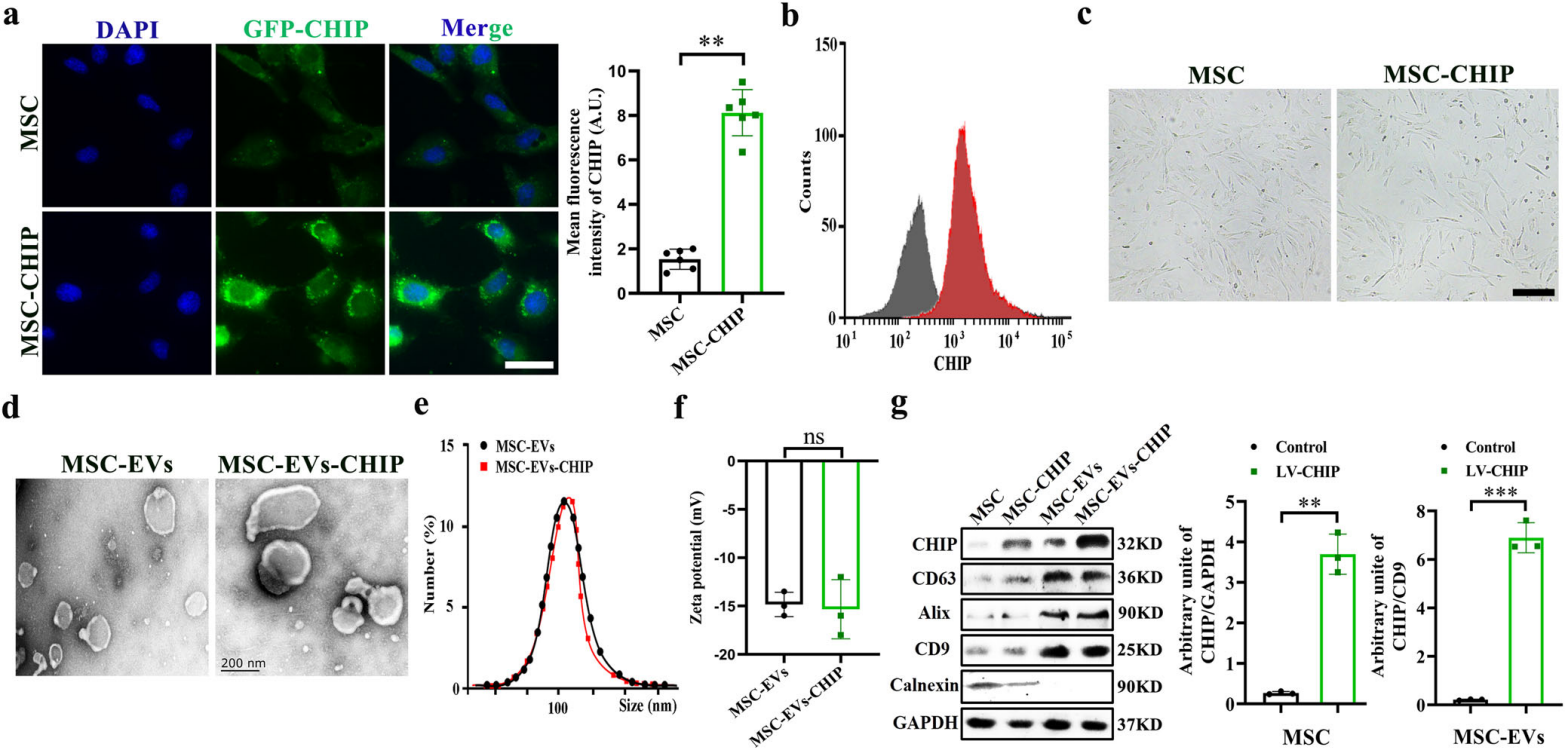
Characteristics of MSC-derived extracellular vesicles with CHIP high-expression. **a** MSCs were transfected with lentivirus encoding the CHIP gene. The expression of CHIP (green) with GFP-tagged in MSCs was determined by immunofluorescence (left). Nuclei were stained with DAPI (Blue). Scale bar: 50 µm. Representative quantification of the mean fluorescence intensity (MFI) of CHIP (*n* = 3) (right). **b** Flow cytometry analysis of CHIP expression in MSCs (left) and representative quantification of the mean fluorescence intensity (MFI) of CHIP (*n* = 3) (right). **c** Morphology of MSCs with lentivirus transfection or non-transfection. Scale bar: 50 µm. **d** Representative transmission electron microscope (TEM) images of MSC-EVs and MSC-EVs- CHIP. Scale bar: 100 nm. **e** The size distribution of MSC-EVs and MSC-EVs-CHIP was measured by dynamic light scattering (DLS). **f** Surface zeta potential of MSC-EVs and MSC-EVs-CHIP (*n* = 3). **g** The expression of CHIP, CD73, CD63, and CD9 in MSCs and MSC-EVs with lentivirus was determined by western blotting (left). Relative values of CHIP/GAPDH and CHIP/CD9 stipe gray (*n* = 3) (right). Data are represented as the mean ± standard error of the mean (SEM). Statistical significance was calculated by a student’s t test. ns non-significance, **P < 0.01, ***P < 0.001.

Umbilical derived MSCs with high purity were isolated from rat according to previous protocols (Supplementary Fig. 1). We next infected MSCs with lentivirus encoding the CHIP gene and selected with puromycin to obtain CHIP high-expressing MSCs, and confocal graph shows that CHIP is mainly expressed in the cytoplasm (Fig. 1a, b). The morphology of MSCs was not affected by high CHIP expression levels (Fig. 1c), lipogenesis and osteogenesis experiment slightly induced stem-cell differentiation compared to non-transfected MSCs (Supplementary Fig. 2). Then MSCs derived EVs were then isolated and purified according to previously described protocols. Transmission electron microscopy (TEM) imaging shows then cup shaped vesicle morphology of MSC-EVs-CHIP (Fig. 1d). And the average particle diameter of MSC-EVs-CHIP measured by dynamic light scattering (DLS) is 108 ± 10.6 nm (Fig. 1e). The zeta potential of MSC-EVs-CHIP was ≈−15 mV (Fig. 1f). Finally, we confirmed the expression of CHIP in MSCs-EVs by western blotting assay. The results showed that CHIP was also highly expressed in engineered EVs containing EVs-associated proteins, such as CD63, CD9, Alix and Calnexin was expressed negatively (Fig. 1g), and GFP signals was also found in EVs-CHIP. These results suggested that the genetically engineered MSC-EVs with high CHIP expression were successfully prepared.

### MSC-EVs-CHIP reduced fibrotic change by inducing Smad2/3 degradation in renal tubular cells

**Fig. 2:**
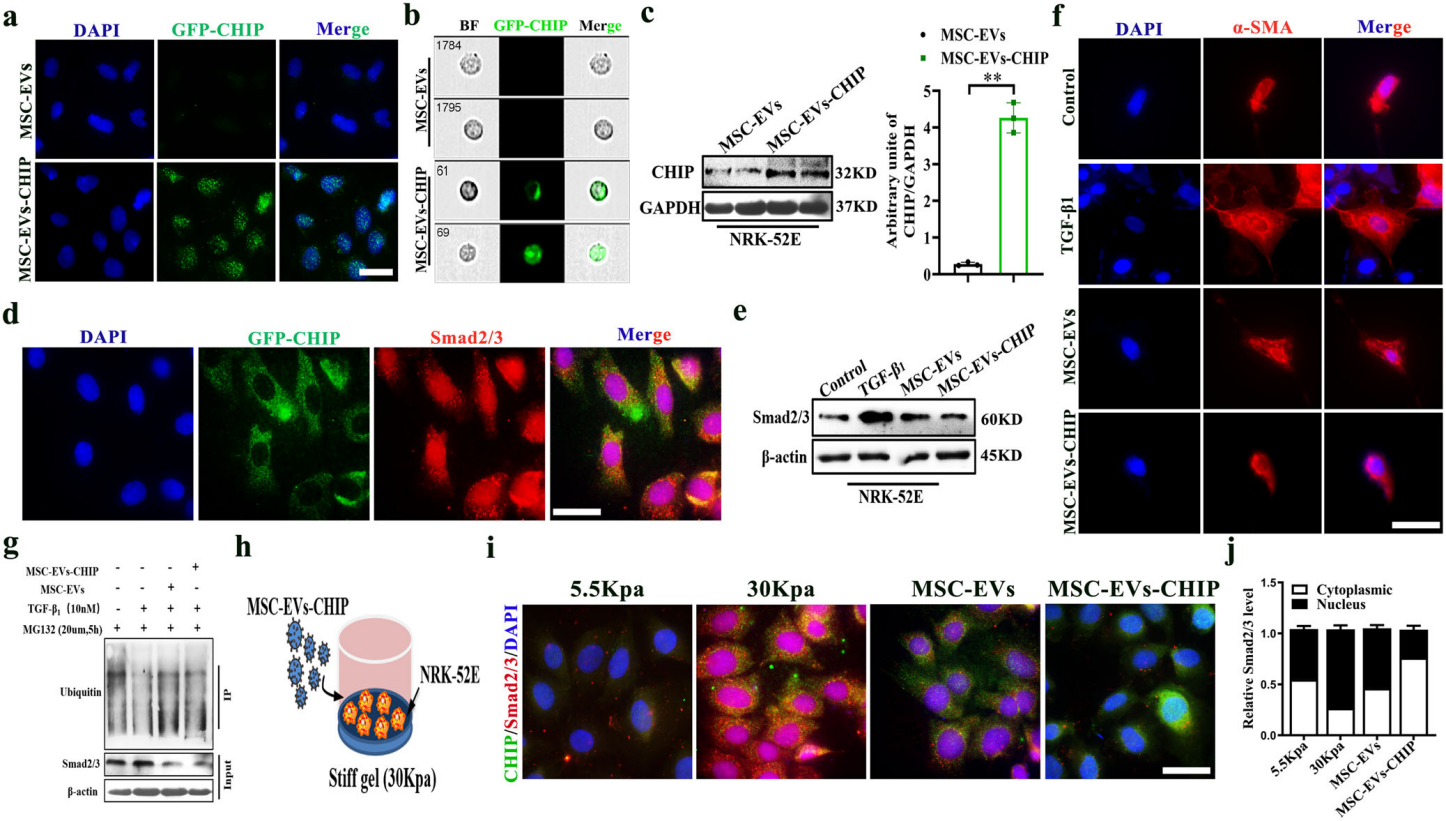
MSC-EVs-CHIP induced Smad2/3 degradation in renal tubular cells. **a** MSC-EVs and MSC-EVs-CHIP were incubated with NRK-52E cells for 12 h. Confocal observed the expression of GFP-CHIP in cells. Nuclei were stained with DAPI (Blue). Scale bar: 25 µm. **b** Flow cytometry shows that NRK-52E cells internalize GFP-CHIP high expression of MSC-EVs. **c** The expression of CHIP in NRK-52E cells was determined by western blotting (left). Representative quantification of CHIP (right) (n = 3). **d** Confocal observed the co-localization of CHIP (Green) with Smad2/3 (Red) of NRK-52E cells. Nuclei were stained with DAPI (Blue). Scale bar: 25 µm. **e** The expression of Smad2/3 in NRK-52E was determined by western blotting. **f** The renal tubular cells (NRK-52E cell line) were pretreated with 10 nM TGF-β_1_ protein in the absence or presence of MSC-EVs and MSC-EVs-CHIP for 24 h. The fibrosis index α-SMA (Red) was observed. Nuclei were stained with DAPI (Blue). Scale bar: 25 µm. **g** Determination of ubiquitin binding on Smad2/3 protein by CO-IP after MSC-EVs and MSC-EVs-CHIP treatment. **h** Diagrammatic of NRK-52E cells were stimulated with 30Kpa stiff gel in the presence of MSC-EVs and MSC-EVs-CHIP for 24 h. **i** The co-localized of CHIP (Green, stained by GFP) with Smad2/3 (Red, stained by Cy3) was observed. Nuclei were stained with DAPI (Blue). Scale bar: 25 µm. **j** Representative quantification of nuclear cytoplasmic ratio of Smad2/3. Data are represented as the mean ± SEM. Statistical significance was calculated by a student’s t-test. ns non-significance, *P < 0.05, **P < 0.01.

Evidence suggests that activation of the Smad2/3 pathway and inflammatory infiltration exacerbate fibrosis in CKD. To test whether MSC-EVs-CHIP induces Smad2/3 degradation and reduce renal interstitial fibrosis, MSC-EVs with GFP-tagged CHIP high expression were incubated with renal tubular cells NRK-52E. As shown by confocal imaging, a high GFP fluorescence signal was observed in NRK-52E (Fig. 2a). Imaging flow cytometry showed that GFP MSC-EVs-CHIP can be internalized by NE-52E cells (Fig. 2b). Western blot experiment confirmed high expression of CHIP in NRK-52E (Fig. 2c). Furthermore, the GFP signal was colocalized with Smad2/3 (Red) in the cells, suggesting that CHIP shifted into the cytoplasm with MSC-EVs (Fig. 2d).

Next, we used fibrosis stimulating factor TGF-β_1_ (10 nM) to induce fibrosis-like change in NRK-52E cells and establish in vitro RIF model. In our experiment, it was found that TGF-β_1_ stimulates α-SMA overexpress and transform into myofibroblasts cells (Supplementary Fig. 3a), MSC-EVs-CHIP significantly inhibited Smad2/3 and fibrotic of TGF-β_1_ incubated cells, as shown by the confocal microphage and western blot (Fig. 2e, f). Additionally, Co-immunoprecipitation observed that MSC-EVs-CHIP promoted ubiquitin molecules expression and decreased the expression of Smad2/3 in TGF-β_1_-incubated cells (Fig. 2g). In order to simulate the mechanical pressure environment in *vivo* by using stiff gel (30Kpa) to stimulate NRK-52E (Fig. 2h, Supplementary Fig. 3b), confocal microscopy also showed that the number of Smad2/3 in nucleus was decreased obviously in NK-52E cells treated with MSCEVs-CHIP relative to that in controls (Fig. 2i), and statistical analysis confirms the results (Fig. 2j). These results demonstrated that MSC-EVs-CHIP could alleviate TGF-β_1_ and stiff gel induced renal tubular cell fibrotic like change by promoting Smad2/3 ubiquitination degradation.

### MSC-EVs-CHIP migrated to the kidney and ameliorated inflammatory infiltration by reduced Smad2/3 accumulation

MSC-EVs-CHIP have been shown to promote Smad2/3 degradation, anti-fibrotic-like change of renal tubular cell, all of which play a critical role in the development and procession of RIF. We further evaluated the therapeutic efficacy of MSC-EVs-CHIP in RIF rat model established by unilateral UUO rat model. First, we assessed the organizational distribution of MSC-EVs-CHIP. CM-DIR labeled MSC-EVs and MSC-EVs-CHIP were injected into rat intravenously for 48 h, then the organ fluorescence was evaluated by PerkinElmer IVIS Lumina II ex vivo imaging. As shown in Figure S4, MSC-EVs and MSC-EVs-CHIP was enriched in liver, hardly enriched in the kidney of control groups, while accumulated in kidney of UUO treated rat (Supplementary Fig. 4). In addition, the fluorescence imaging of the kidney tissue of RIF-rat receiving MSC-EVs-CHIP exhibited significant CM-DIR signals relative to the control (Supplementary Fig. 5a). Western blotting analysis further showed that MSC-EVs treatment resulted in a high CHIP level in kidney tissue (Supplementary Fig. 5b). These data suggested that under RIF pathological conditions, MSC-EVs-CHIP have an ability to target the injury kidney.

**Fig. 3:**
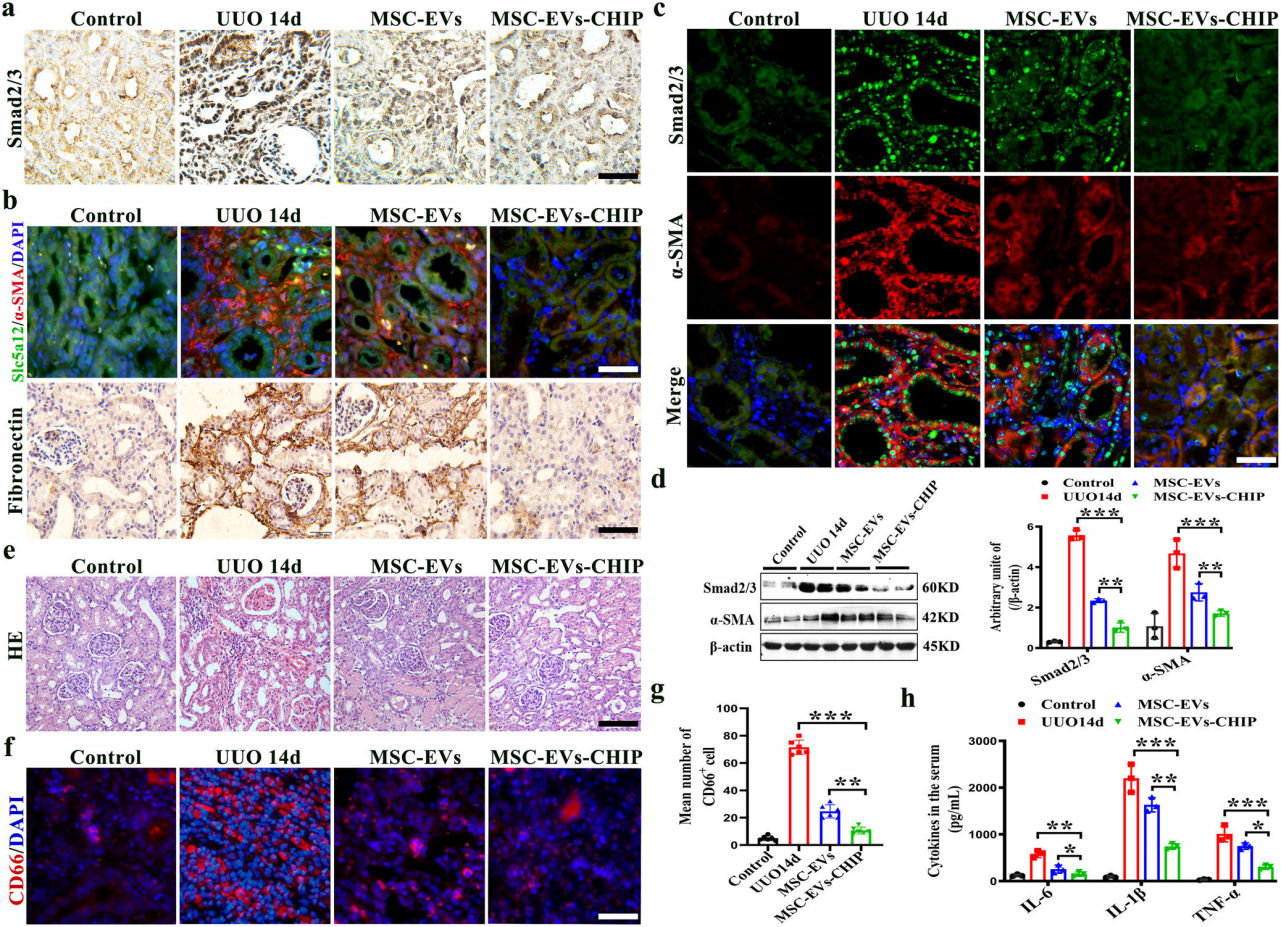
MSC-EVs-CHIP inhibited Smad2/3 and reduced inflammatory infiltration. **a** RIF was established by ureteral ligation of SD rat for 14 days, and intravenously injected with MSC-EVs and MSC-EVs-CHIP for three times in 7 days. Representative images of Smad2/3 in renal interstitial were demonstrated by immunohistochemistry (*n* = 3). Scale bar: 100 µm. **b** The localized of fibrosis marker α-SMA (Red) with renal tubular cell marker Slc5a1 (Green) in renal was showed by immunofluorescence (left). Nuclei were stained with DAPI (Blue). And representative images of Fibronectin were demonstrated by immunohistochemistry. Scale bar: 100 µm. **c** The co-localized of Smad2/3 (Green) with α-SMA (Red) in renal was showed by immunofluorescence (left). Nuclei were stained with DAPI (Blue). Scale bar: 100 µm. **d** The expression of Smad2/3, α-SMA and β-actin in kidney was determined by western blotting (left). Relative values of Smad2/3 and α-SMA stipe gray (*n* = 3) (right). **e** Representative images of HE staining of renal inflammatory infiltration. Scale bars: 100 µm. **f** The representative quantitation of Inflammatory infiltration was demonstrated by CD66 (Red) immunofluorescence staining. Nuclei were stained with DAPI (Blue). Scale bar: 100 µm. **g** Representative number of CD66 (Red) positive cell (*n* = 6). Nuclei were stained with DAPI (Blue). Scale bar: 100 µm. **h** The production of cytokines (IL-6, IL-1β, TNF-α) in the rat serum was measured using enzyme-linked immunosorbent assay (ELISA) kits (*n* = 3). Data are represented as the mean ± SEM. Statistical significance was calculated by a student’s t test. ns non-significance, *P < 0.05, **P < 0.01, ***P < 0.001.

We next investigated whether MSC-EVs-CHIP promote smad2/3 degradation and inhibits inflammation in the kidney of RIF model. Rats were raised for 7 days after unilateral ureteral obstruction, and were then intravenously treated with MSC-EVs or MSC-EVs-CHIP (2 mg per rat/every three days). After one week, we performed immunofluorescence analysis of the renal tissue. In UUO rats, Smad2/3 is highly expressed in the nucleus of renal tubular epithelial cells, and fibrosis-related proteins (Collagen I, α-SMA) are significantly increased (Supplementary Fig. 6). While the renal of rat receiving MSC-EVs-CHIP, we observed a significantly reduced expression of Smad2/3 in renal tubular nucleus compared to MSC-EVs treatment, suggesting the downregulation of Smad2/3 in the renal tissue of RIF rat (Fig. 3a). In addition, the fibrosis related indicators α-SMA and Fibronectin in the kidneys of RIF rats receiving MSC-EVs-CHIP were significantly reduced (Fig. 3b). We further observed the reduced Smad2/3 accumulation and tubular cells fibrotic like change in the renal of MSC-EVs-CHIP-treated rat by immunofluorescence analysis, suggesting that MSC-EVs-CHIP can significantly rescue RIF pathology by promoting Smad2/3 degradation and recovering normal homeostatic processes (Fig. 3c, d).

Given the critical role of inflammation in the progression of RIF, its effect on inflammation in renal was also evaluated after MSC-EVs-CHIP intervene. Persistent mechanical damage can stimulate the inflammatory response in tubular cells, especially the CD66 positive neutrophil accumulation, as shown in the HE staining and immunofluorescence (Fig. 3e–g). Encouragingly, the induced inflammation and cytokine (IL-6, IL-1β, TNF-α) release was remarkably inhibited by MSC-EVs-CHIP treatment (Fig. 3h). These data suggested that CHIP overexpression mediated by MSC-EVs inhibited Smad2/3 and decreased fibrosis index, further remitted the inflammation in RIF-rat.

### MSC-EVs-CHIP reduced interstitial collagen deposition in UUO model

**Fig. 4:**
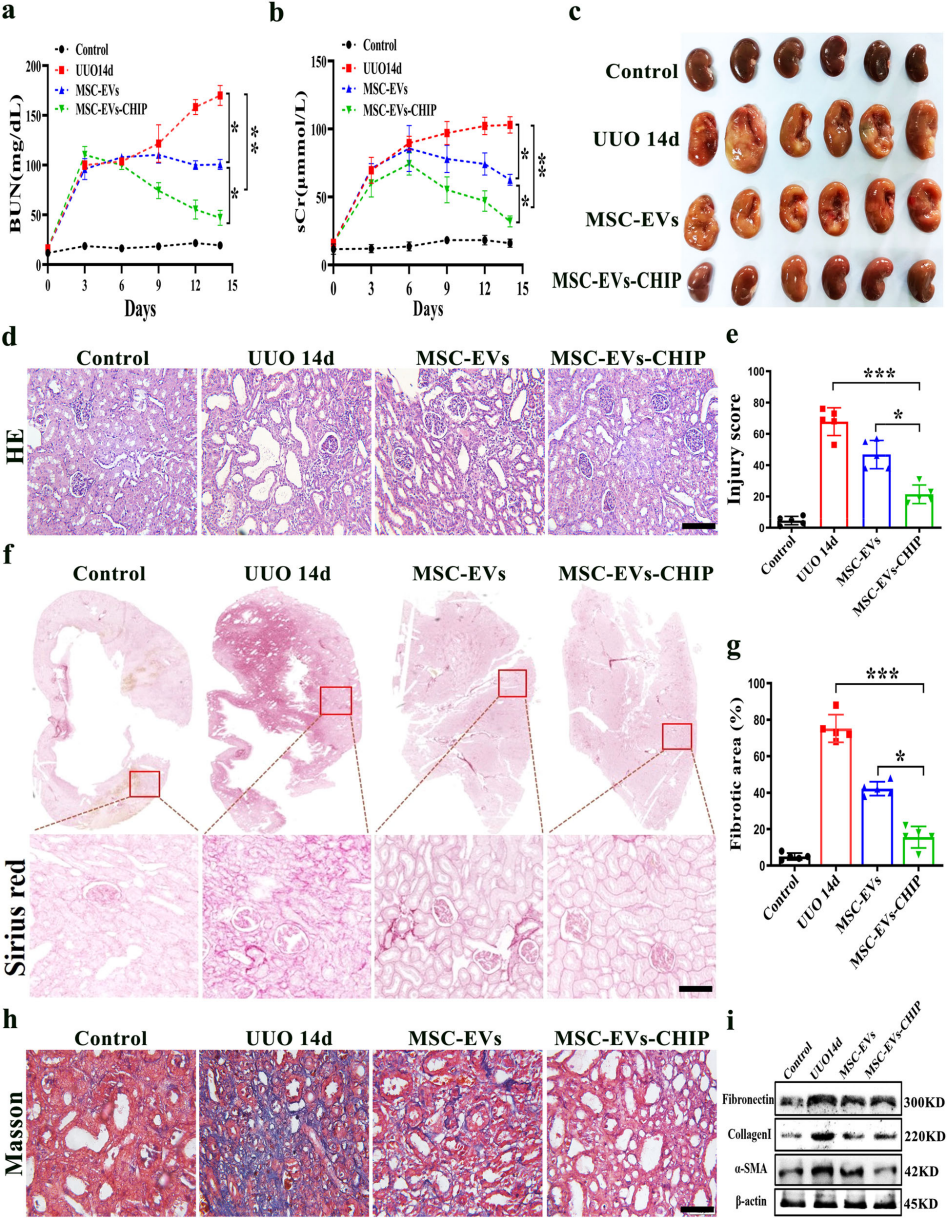
MSC-EVs-CHIP reduced collagen deposition in UUO model. **a** Effects of MSC-EVs-CHIP on serum urea nitrogen and **b)** creatinine. **c** Representative images of renal tissues established by UUO rats (*n* = 6 per group) that received different treatments including sham, PBS, MSC-EVs (10 mg/kg of body weight), MSC-EVs-CHIP (10 mg/kg of body weight). **d** Representative images of HE staining in MSC-EVs and MSC-EVs-CHIP group (*n* = 3). Scale bars: 100 µm. **e** The quantification of tubular injury based on HE staining (*n* = 5). **f** Representative images of Sirius red staining (*n* = 3). The bottom was a higher magnification of the boxed region. Scale bars: 1 mm (top) and 100 µm (bottom). **g** The quantification of fibrotic area based on Sirius red staining (*n* = 5). **h** Representative images of Masson trichrome staining on renal tissues sections (*n* = 3). Scale bars: 100 µm. **i** Western blotting analysis of Fibronectin, α-SMA, and Collagen I in kidney tissues.

Inject the prepared MSC-EVs-CHIP and MSC-EVs into the tail vein of the UUO model, and collect large mouse notebooks from each group on the 15th day. The biochemical analyzer detected renal function indicators such as creatinine and urea nitrogen, and found that the MSC-EVs-CHIP group significantly downregulated creatinine and urea nitrogen compared to the MSC-EVs group, significantly improving renal function (Fig. 4a, b). The appearance of kidneys in the UUO group significantly larger and lighter in color, while the kidney tissue significantly returned to control after MSC-EVs-CHIP treatment (Fig. 4c). Further through the HE staining results of renal tissue pathology showed that the UUO injury group had an increase in tubular vacuolar degeneration, a significant thickening of the glomerular basement membrane, and a tissue damage score of 70%. Compared with the MSC-EVs group, the MSC-EVs-CHIP group restored normal renal tissue structure after intervention, and reduced tubular vacuolar degeneration. The damage score was reduced to 18% (Fig. 4d, e). The results of Sirius red staining and Masson staining showed significant fibrosis in UUO renal tissue. The change area reached 64%, while the MSC-EVs-CHIP group showed a significant reduction in renal interstitial collagen fibers and a significant decrease in fibrosis degree after treatment, which was better than the MSC-EVs treated group alone (Fig. 4f-h). Extract kidney tissue proteins from each group for Western blot detection, displaying fibrosis related proteins Fibronectin, Collagen I, and α-SMA were significantly reduced (Fig. 4i). The above research results indicate that high expression of CHIP enhances MSC-EVs in improving renal function and reducing renal interstitial collagen.

### In vivo renal-targeting and antifibrotic therapeutic effect of SPION-EVs

**Fig. 5:**
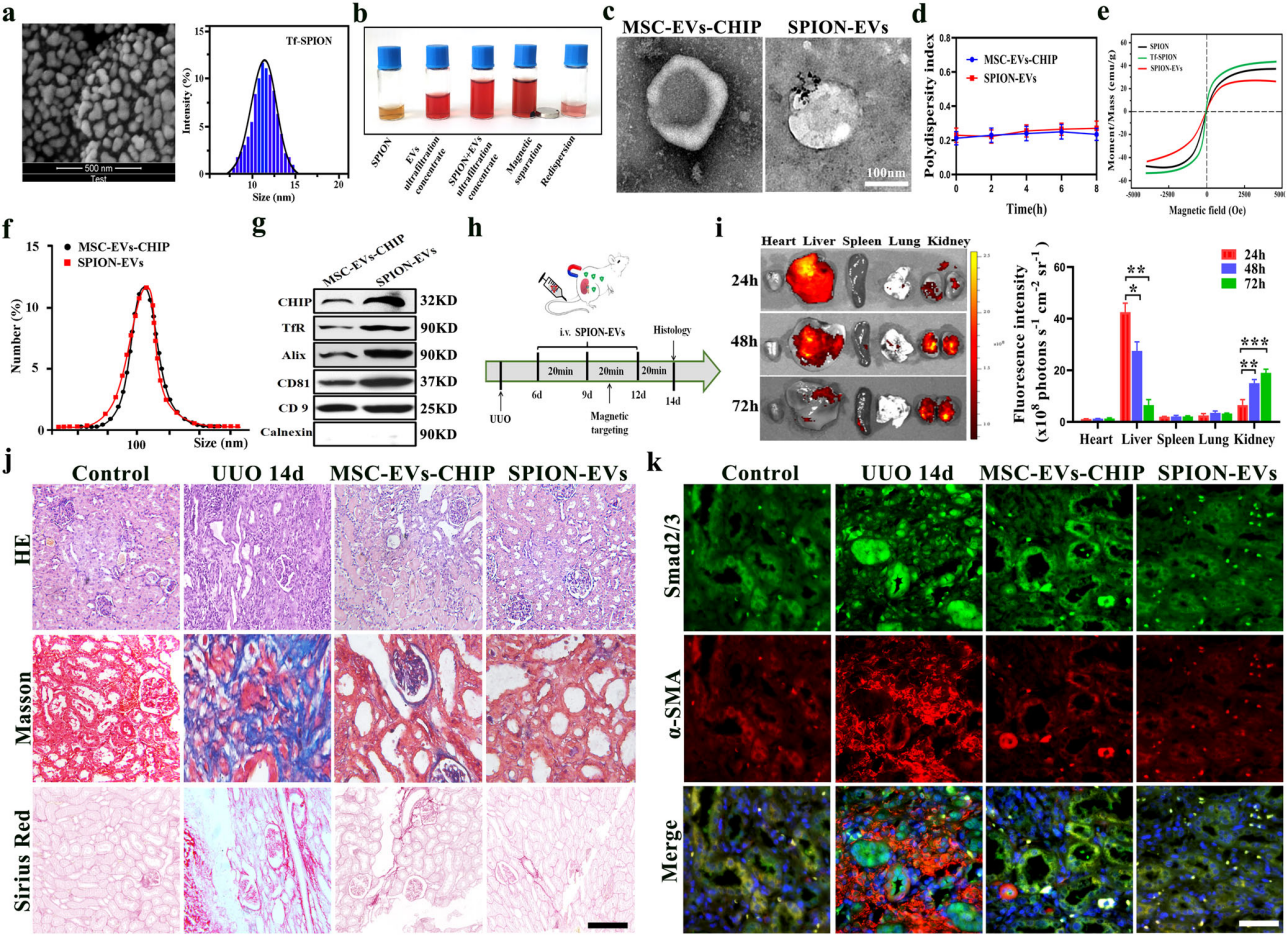
In *vivo* renal-targeting and antifibrotic therapeutic effect of SPION-EVs. **a** TEM and dynamic light scattering (DLS) detect the graphic and nanoparticle size of SPION. Scale bar: 500 nm. **b** Separation and preparation of SPION-EVs flowchart. **c** Representative TEM images of MSC-EVs-CHIP and SPION-EVs. Scale bar: 100 nm. **D** Stability analysis of SPION-EVs using polymer dispersibility index (PDI). **e** Hysteresis loop analysis of SPION-EVs. **f** DLS detect the nanoparticle size of SPION-EVs. **g** The expression of CHIP, TfR, Alix, CD63, and CD9 in MSC-EVs-CHIP and SPION-EVs was determined by western blotting. **h** Schematic diagram of the experimental design. Briefly, rats were concurrently treated with MSC-EVs-CHIP and SPION-EVs (2 mg) every 3 days after renal UUO injury 7 days and were euthanized at 7 days after disease induction. **i** Intravenously injected with CM-DIR-labeled MSC-EVs-CHIP and SPION-EVs for 24, 48,72 h in UUO model. Ex vivo fluorescence images of CM-DIR-labeled EVs in major organs (left). Fluorescence intensity per gram of tissue in heart, liver, spleen, lung and kidney (right) (n = 3). **j** The role of SPION EVs in anti-fibrotic were demonstrated by HE and Masson staining, Sirius red staining (n = 3). Scale bar: 100 µm. **k** The expression of Smad2/3 (Green) with α-SMA (Red) after MSC-EVs-CHIP and SPION-EVs treatment in renal was showed by immunofluorescence. Nuclei were stained with DAPI (Blue). Scale bar: 100 µm. Data are represented as the mean ± S.E.M. Statistical significance was calculated by a student’s t test. ns, non-significance, *P < 0.05, **P < 0.01, ***P < 0.001.

In order to further enhance the anti-fibrotic effect of MSC-EVs, we used the SPION with surface modified transferrin (Tf) to combine with the transferrin receptor on the surface of MSC EVs CHIP for engineering modification to enhance its ability to target the injured site. Scanning electron microscope observation shows that the size of the nanospheres is about 5 nm, and dynamic light scattering shows that the size of the nanoparticle is about 10 nm (Fig. 5a). The superparamagnetic modification of transferrin binds to the transferrin receptor on the surface of the MSC-EVs-CHIP membrane after co incubation at 4 °C for 4 h, and SPION-EVs are collected and dissolved in PBS by external magnetic field adsorption (Fig. 5b). The elliptical vesicle structure combined with multiple SPION was observed by transmission electron microscopy (Fig. 5c). Analyze the stability and magnetic characteristics of SPION-EVs in aqueous solutions using hysteresis loops and PDI polymer dispersion index (Fig. 5d, e). The average particle size of SPION-EVs measured by dynamic light scattering (DLS) is 115 ± 11.3 nm, compared to MSC-EVs-CHIP, the average particle diameter increases by about 7 nm, indicating that SPION not obvious change the size of extracellular vesicles (Fig. 5f). The zeta potential of SPION-EVs was ≈−16 mV (Supplementary Fig. 7). Western blot was used to detect the expression of surface positive marker proteins CD9, CD81, Alix and negative marker Calnexin in SPION-EVs, and strong positive expression of CHIP and transferrin receptor TfR (Fig. 5g). This indicates that we have successfully prepared engineered SPION-EVs.

Construct the renal fibrosis treatment model using prepared engineered SPION-EVs under external magnetic field intervention (Fig. 5h). By using a small animal live imaging system, it was shown that under the action of an external magnetic field, the signal intensity of DIR labeled SPION-EVs was enriched by targeting the liver to the kidneys (Fig. 5i). The pathological tissue showed a decrease in tubular vacuolar degeneration and a return to normal structure in the SPION-EVs intervention group. Sirius red and Masson staining confirmed that compared to the MSC-EVs -CHIP group, the SPION-EVs group showed a decrease in interstitial collagen fibers and a significant decrease in fibrotic areas (Fig. 5j). Immunofluorescence showed that SPION-EVs significantly inhibited the nuclear uptake of Smad2/3 in renal tubular cells and decreased α-SMA was superior to the MSC-EVs-CHIP group (Fig. 5k). The above results indicate that the targeted enrichment of engineered SPION-EVs under magnetic field significantly enhances the anti-fibrotic effect.

### SPION-EVs attenuated renal interstitial fibrosis in the DKD model

**Fig. 6:**
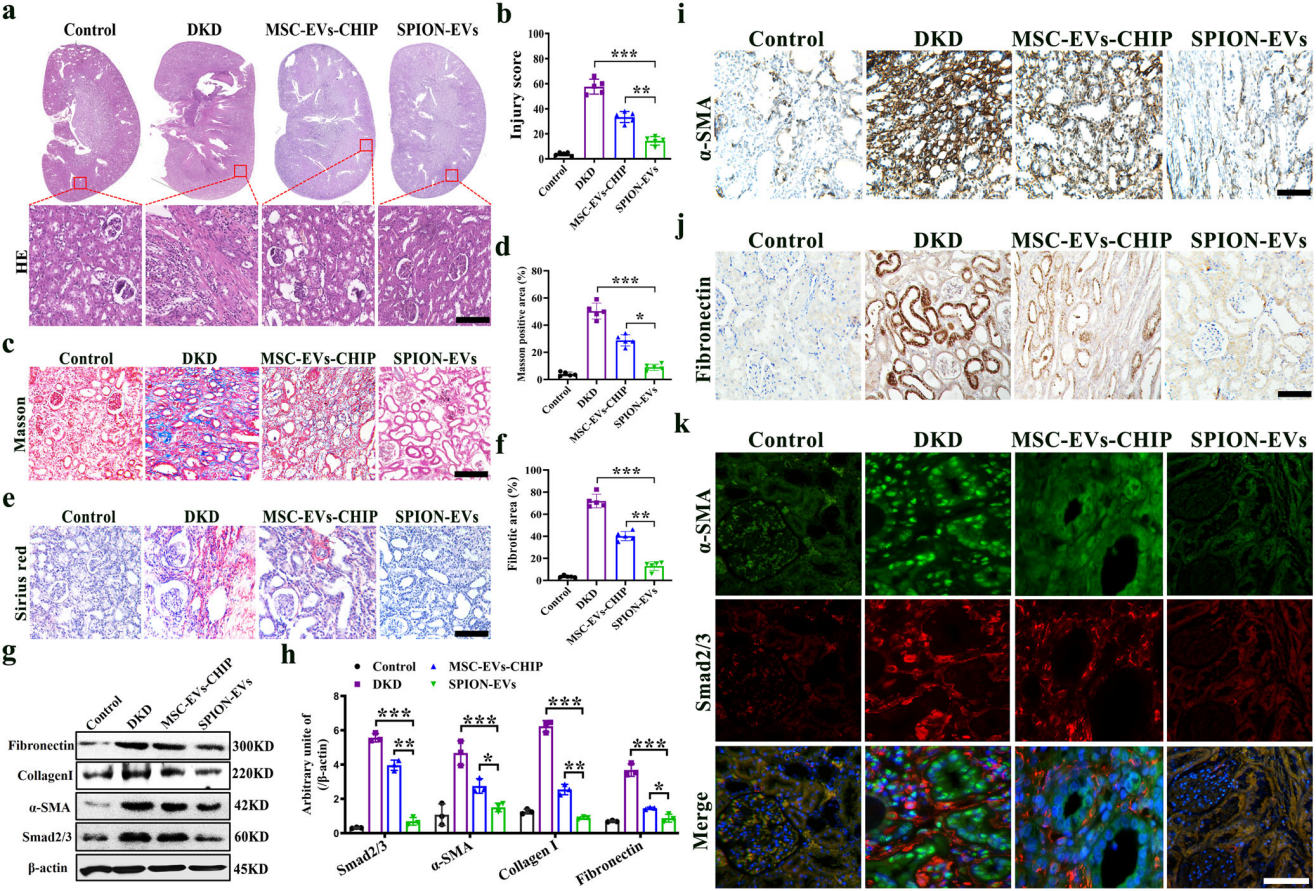
The anti-fibrotic effect of SPION-EVs in DKD model. **a** Representative image of HE staining with MSC-EVs-CHIP and SPION-EVs treatment in DKD model (*n* = 3). The bottom was a higher magnification of the boxed region. Scale bars: 1 mm (top) and 100 µm (bottom). **b** The quantification of tubular injury score based on HE staining (*n* = 5). **c** Representative images of Sirius red staining (*n* = 3). Scale bars: 100 µm. **d** The quantification of Masson positive area based on Masson staining (n = 5). **e** Representative images of Sirius red staining (*n* = 3). Scale bars: 100 µm. **f** The quantification of fibrotic area based on Sirius red staining (*n* = 5). **g** The expression of Smad2/3, α-SMA, Fibronectin and Collagen I in DKD model was determined by western blotting (left). **h** Relative values of Smad2/3, α-SMA, Fibronectin and Collagen I stipe gray (*n* = 3) (right). **i** Representative images of α-SMA were demonstrated by immunohistochemistry. Scale bar: 100 µm. **j** Representative images of Fibronectin in DKD model were demonstrated by immunohistochemistry. Scale bar: 100 µm. **k** The expression of Smad2/3 (Green) with α-SMA (Red) after MSC-EVs-CHIP and SPION-EVs treatment in DKD model was showed by immunofluorescence. Nuclei were stained with DAPI (Blue). Scale bar: 100 µm. Data are represented as the mean ± SEM. Statistical significance was calculated by a student’s t test. ns, non-significance, *P < 0.05, **P < 0.01, ***P < 0.001.

**Fig. 7:**
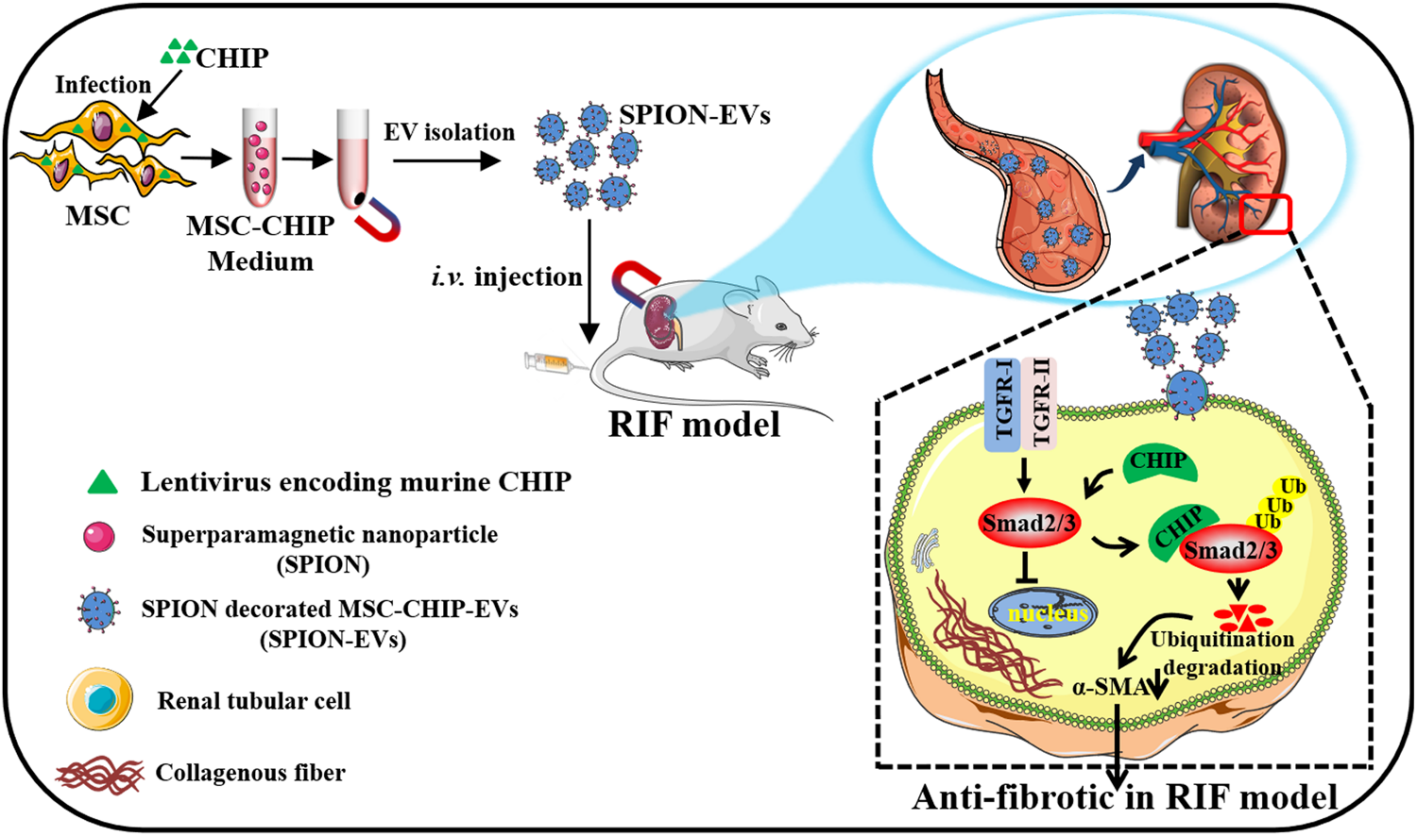
Schematic illustrations of engineered extracellular vesicle as novel nanotherapeutics against renal fibrosis. A platform for RIF therapy using SPION decorate nanosized mesenchymal stem cells-derived extracellular vesicles with carboxyl terminus of Hsc70-interacting protein (CHIP) high-expression (SPION-EVs) is developed. SPION-EVs significantly enrichment of renal injury sites and induces Smad2/3 degradation of renal tubular cells, which alleviates fibrosis and inflammation, accompanying with rescued collagen deposition in RIF.

To further prove that SPION-EVs is a promising nanoplatform for the delivery of CHIP and treatment of renal fibrosis, we constructed another renal interstitial fibrosis model in diabetic kidney disease (DKD) rat. The DKD injury model was established by feeding high-fat combined with streptozotocin (STZ) for 18 weeks, intervened by tail vein injection of SPION-EVs. Firstly, we assessed the alleviation of SPION-EVs against interstitial fibrosis in DKD rat. As expected, histological analysis of kidney sections at 24 weeks after STZ injection revealed significant tubulointerstitial damage, including tubular atrophy, cast formation, infiltrates of leukocytes, and fibrosis, all of which were markedly ameliorated by SPION-EVs treatment (Fig. 6a, b). To fully assess the efficacy of SPION-EVs, kidney sections were subjected to Masson and Sirius red staining to determine the fibrosis index and stained with anti-α-SMA/Fibronectin antibody for the detection of fibrosis-like tubules. The results demonstrated that the renal fibrosis was reduced in DKD rat treated with SPION-EVs (Fig. 6c–f), and reduction of α-SMA, Collagen I, and Fibronectin deposition in renal tissues of SPION-EVs treated DKD rat (Fig. 6g–j). Moreover, immunofluorescence shows that SPION-EVs can significantly inhibit the nuclear expression of Smad2/3 in renal tubules stimulated by high glucose environment and reduce α-SMA (Fig. 6k). These data suggested that SPION-EVs could attenuate renal fibrosis and retarding the progress of DKD (Fig. 7).

### Assessment of toxicity and inflammation in SPION-EVs–treated rat model

We next examined the concentrations of hepatic enzyme such as alanine aminotransferase and aspartate aminotransferase in the serum of SPION-EVs treated rats showed no obvious differences from those of phosphate-buffered saline (PBS) treated rats (Supplementary Fig. 8a). Similarly, SPION-EVs and MSC-EVs-CHIP group toxicity on the major organs, including the heart, liver, spleen and lung. The histological analysis showed no evidence of in vivo toxicity of SPION-EVs (Supplementary Fig. 8b). Consistent with previous studies that engineered cell-derived EVs are biocompatible and not significantly toxic.

## Discussion

In this study, we reported a method for manufacturing engineered SPION-EVs and evaluated the feasibility, safety, and effectiveness of MSC-EVs with CHIP overexpressed in treating renal interstitial fibrosis in UUO and DKD models. Impressively, provide packed CHIP protein into MSC-derived EVs by genetic engineering with lentivirus transfection (Fig. 1), then through nanomaterials SPION decorated MSC-EVs with CHIP overexpressing target and enrich the damaged kidneys, resulting in notable renal protective effects, specifically by promoting Smad2/3 ubiquitination and degradation to reverse the transformation of renal tubular cells into myofibroblast. Our findings not only demonstrate the role of engineered MSC extracellular vesicles in the anti-fibrotic activity, but also as a new renal fibrosis therapeutic agent and target delivery nanoplatform. In a word, engineered SPION-EVs constitute an effective nanotherapeutic for the renal fibrosis treatment.

Over the past several years, the clinical studies of RIF therapies have concentrated on antifibrotic, including anti-fibrosis antibodies and YAP inhibitors^24,25^. Unfortunately, these treatment strategies have not achieved good results and were not significantly reduce fibrosis in clinical tests. Furthermore, the pathogenesis of RIF is complex and includes inflammation, tubular injury, oxidative stress and metabolic dysfunction^26^. There is increasing evidence that Smad2/3 ubiquitination and degradation in renal interstitial is a promising treatment method for CKD therapy^27^. we developed an SPION decorated engineered MSC-EVs platform by CHIP expression to reduced renal fibrosis and inflammatory infiltration in RIF rats. In vitro, we observed that MSC-EVs-CHIP inhibit Smad2/3 expression in rat renal tubular epithelial cells and relieved cells myofibroblast change induced by TGF-β_1_, and inhibited inflammasome activation. In UUO and DKD rat model, MSC-EVs-CHIP promoted Smad2/3 ubiquitination and degradation, further alleviated tubular cells fibrosis and inflammation.

CHIP as an E3 Ubiquitin ligase, that induces protein folding and degradation balance by upregulating ligase expression or activation^28^. And which is an important component of the protein quality control system, preventing and treating diseases related to protein metabolism disorders^29^. CHIP protein could be used as a biological drug for the treatment of RIF. However, due to the distribution of metabolism in *vivo*, and poor renal target efficacy, the poor stability, lead to the CHIP protein can hardly be used in clinical practice. Moreover, we verified the low enrichment of CHIP protein in kidney tissue by intravenous injection in the RIF rat model. According to our previous works on genetically engineering decorated MSC-EVs for delivery of the key protein molecules, which have packed CHIP protein into MSC-EVs by genetic engineering with lentivirus transfection, then use SPION decorated MSC-EVs-CHIP that resolved the challenges for enrichment and renal-target.

Mesenchymal stem cell derived extracellular vesicles as cell-free long-distance delivery systems have displayed apparent advantages, for instance high injury tissue target ability, tissue repairment capacity and low immunogenicity, which have been applicated in clinical therapies^30^. It has reported that MSC-EVs have strong tissues target capacity to realize drug delivery^31^. Our previous research confirmed that MSC-EVs attenuate renal interstitial fibrosis through the kinase ubiquitin system CK1δ/β-TRCP mediated YAP ubiquitination and degradation^24^. MSC-EVs could to some extent reach the site of damaged kidney tissue and exert the effective on tissues fibrosis, however, the fluorescence statistical distribution of MSC-EVs labeled with DIR membrane dyes, we found that MSC-EVs were mainly concentrated in the liver and lungs. This has prompted us to think deeply about the need to enhance the targeted enrichment of extracellular vesicles through engineering transformation.

Superparamagnetic iron oxide nanoparticles (SPION) have been widely used in disease therapy for excellent superparamagnetism property. The conjugation of SPIONs with extracellular vesicles has many advantages, including magnetic targeted functionalization, magnetic thermotherapy, and delivery of anti-fibrosis agents^32^. Furthermore, in order to reduce the uptake of MSC-EVs in the liver and lungs after tail vein injection, nanomaterials SPION modified MSC-EVs were used to enhances the penetration and enrichment of MSC-EVs to renal under external magnetic field.

In conclusion, we have exploited a therapeutic strategy for renal fibrosis using nanomaterials SPION decorated MSC-EVs-CHIP to increase target the concentration of CHIP in renal tissue. We have constructed a platform for targeted delivery of CHIP by using MSC-EVs and highlighted the potential of SPION-EVs as a promising nanotherapeutic for renal interstitial fibrosis treatment. Our platform may provide potential for clinical application in the future owing to the advantages of engineered MSC-EVs and the novel inducing Smad2/3 protein degradation strategy in RIF treatment.

## Methods

### Ethics statement for human samples and animal models

Written informed consent was obtained from healthy donors, and discarded human umbilical cord tissues were acquired according to the approved protocol of the Institutional Review Board (IRB) at the Affiliated Hospital of Jiangsu University. And conducted in accordance with ethical principles of the World Medical Association Declaration of Helsinki. All animal experimental protocols were approved by the Animal Care and Use Committee of Jiangsu University (Approval number: 2020280). All animal experiments were performed in accordance with the associated relevant guidelines and regulations for working with live vertebrate animals.

### Animals

Sprague-Dawley rat (male, 6–8 weeks old, 180–200 g) were purchased from the Experimental Animal Center of the Jiangsu University. And db/db mice (female, 6–8 weeks old, 18–22 g) were purchased from the Cavens Experimental Animal Co., Ltd (Changzhou). They were provided free access to pellet food and kept water in plastic cages at 20 ± 2 °C and kept on a 12 h light/dark cycle in specific pathogen-free facilities. The experimental endpoints the animals were anesthetized euthanasia with pentobarbital sodium (Sprague-Dawley rat: 50 mg/kg, db/db mice: 30 mg/kg, Sigma-Aldrich) by intraperitoneal injection. Kidneys were fixed with 4% formaldehyde, embedded in paraffin, and sectioned to 4 μm thickness.

### Cell culture

The rat renal tubular cells line NRK-52E was purchased from National Collection of Authenticated Cell Cultures. NRK-52E cells were maintained in DMEM (GIBCO, USA) containing 10% fetal bovine serum, 100 U mL^−1^ penicillin, and 100 mg mL^−1^ streptomycin in 5% CO_2_ at 37 °C.

Mesenchymal stem cells were isolated from human umbilical cord according to a previously described method. In brief, take the umbilical cord from a full-term newborn, wash with PBS and cut off the arteries and veins. Cut it into 2 mm sized tissue blocks and stick them on a 6-well plate with an interval of 5 mm. Add basic DMEM (GIBCO, USA) medium containing 10% fetal bovine serum and 100 U mL^−1^ penicillin, and 100 mg mL^−1^ streptomycin in 5% CO_2_ at 37 °C. Thereafter, the medium was refreshed every 3 days and continuous culture until the purity of mesenchymal stem cells reached 85%.

### Extraction and Purification of MSC-EVs-CHIP

A lentivirus vector encoding murine CHIP was purchased from genepharma (Suzhou, China). Mesenchymal stem cells were transfected with lentivirus encoding CHIP gene. When the transfection efficiency reached 80% measured by western blotting and flow cytometry analysis. The EVs in medium and FBS were depleted by ultracentrifugation. The medium was replaced and collected after 48 h. MSC-EVs were isolated and purified by ultracentrifugation^33^. In brief, the medium was centrifuged at 800 g for 10 min, 2000g for 10 min, 10 000 g for 30 min, 100,000 g for 70 min at 4 °C, to obtain MSC-EVs. The specific marker expression of CD63, CD9, CD81, and the CHIP expression was measured by western blotting.

### Biological characterization analysis of MSC-EVs-CHIP

Prepared MSC-EVs and MSC-EVs-CHIP samples were pipetted onto a 300 meshes copper and incubated for 3 min. 20 µL of 1% uranyl acetate was pipetted onto the mesh copper and incubated for 1 min, washed and dry for TEM. The images were acquired using transmission electron microscope. MSC-EVs size determination by dynamic light scattering (DLS), in brief the volume of MSC-EVs and MSC-EVs-CHIP samples was increased to 1 mL with PBS and loaded into a quartz cuvette. The size distribution of EVs was measured by dynamic light scattering at 25 °C. Nanometer tracking analyzer detected its zeta potential and nanoparticle size.

### Renal interstitial fibrosis model construction and treatment

RIF was established in Sprague-Dawley rats, anesthetize with inhalation of 2% isoflurane, expose the left ureter through a lateral incision, and ligated with double straps and surgical thread. The rats were randomly assigned into five experimental groups: Control, UUO, MSC-EVs, and MSC-EVs-CHIP, SPION-EVs. Rats were ureteral obstruction for 2 weeks, and intravenously injected with MSC-EVs, MSC-EVs-CHIP and SPION-EVs (2 mg per rat/every three days). After 2 weeks of treatment, the renal and blood serum were collected and analyzed renal function. The biodistribution of MSC-EVs-CHIP and SPION-EVs was performed a PerkinElmer IVIS Lumina II. In brief, MSC-EVs-CHIP and SPION-EVs was labeled with CM-DIR, and the free CM-DIR was washed thrice with cold PBS by ultracentrifugation for 3 times with cold PBS. Then, the CM-DIR-labeled EVs were injected intravenously into rat for 12 h and the CM-DIR signal in various tissues by PerkinElmer IVIS Lumina II.

### RNA extraction and RT-qPCR

Trizol reagent was applied to extract total RNA from cultured cells or tissues in strict accordance with the instructions provided on the kit, followed by the determination of RNA concentration. mRNA was reverse-transcribed to cDNA and subjected to quantitative PCR, which was performed with the BioRad CFX96 Real-Time PCR Detection System (BioRad, USA). mRNA expression was compared using the 2^−ΔΔCt^ relative quantification method, GAPDH was used as the endogenous control.

### Western blot analysis

Renal tubular cells and kidney tissues were lysed using lysis buffer and the obtained protein lysates were quantitated by BCA assay, then degenerated at 100 °C for 5 min. Proteins were resolved by sodium dodecyl sulfate-polyacrylamide gel electrophoresis, transferred onto polyvinylidene fluoride membranes and blocked with 5% bovine serum albumin. Membranes were immunoblotted with primary rabbit polyclonal antibodies to alpha-smooth muscle actin (α-SMA; 1:500, AB32575), Mothers against decapentaplegic homolog 2 (Smad2/3; 1:1000, CST3102), Fibronectin (FN, 1:1000, CST26836), Collagen I (Collagen I, 1:1000, CST72026) and incubated overnight at 4 °C with constant shaking. Next, the primary antibody-incubated membranes were washed five times with washing buffer and incubated with HRP-coupled secondary antibody for 1 h at room temperature. Protein bands were visualized by western blotting. Band intensity was quantified using the Image J software. The relative expression of the target protein was normalized to the band intensity of β-actin. We have included original western blot chemiluminescent images with corresponding light micrographs showing molecular weight markers for all western blots in Supplementary Fig. 10.

### Histologic analysis

Renal tissues samples were fixed in 10% formalin solution, embedded in paraffin and sliced into 5μm thick sections. After deparaffinization and rehydration, sections were stained with hematoxylin and eosin (HE), Sirius red and Masson. The area at the junction of the cortex and medulla in the renal section was selected for histological analysis. HE staining was used to assess tubular injury, Sirius red and Masson staining was performed to evaluate the deposition of collagenous fibers in the renal interstitium. The positive area (red) of Sirius red and (blue) Masson staining was quantified using Image J.

### Immunohistochemistry and Immunofluorescence

Paraffin-embedded tissues were heat-fixed, deparaffinized, rehydrated, antigen retrieved, and subsequent process. First, renal tissue sections were heat-fixed for 2 h at 60 °C and deparaffinized in xylene for 30 min, then rehydrated in 100%, 75%, and 50% ethanol respectively for 10 min. Antigen retrieval was performed using 10 × 10^−3^ M citrate antigen retrieval solution under high pressure for 30 min. The sections were incubated with 3% H_2_O_2_ for 10 min in the dark, blocked with 10% BSA for 1 h and stained with Smad2/3 and Fibronectin antibodies overnight at 4 °C, and washed three times with PBS. 50 µL HRP-labeled goat anti-mouse/rabbit Ig mixture was added to sections for 30 min and washed with PBST thrice. 50 µL DAB reagent was added for color reaction and restained with hematoxylin for 30 s. Finally, the sections were soaked in 50%, 75%, 100% ethanol, and xylene for 10 min respectively to dehydrate. The images of samples were acquired using Leica fluorescence optical microscope.

The renal sections after deparaffinized and rehydrated, then stained with Smad2/3, α-SMA, CD66, and CHIP, Slc5a12 antibodies overnight at 4 °C, and washed thrice times with PBS. The sections were stained with DAPI for confocal laser scanning microscopy (GE, USA). NRK-52E cells were washed twice with cold PBS. Then cells were fixed in 4% paraformaldehyde (PFA) for 10 min, blocked with 10% BSA for 1 h at room temperature, and stained with CHIP, Smad2/3, and α-SMA antibodies overnight at 4 °C. The cells were then washed thrice times with cold PBS, and stained with DAPI for confocal laser scanning microscopy (GE, USA).

### Statistical analysis

All the experiments were randomized and blinded. All studies were performed in at least three independent experiments with each experiment including triplicate sets in vitro, or six animals per group in vivo. All data are presented as the mean ± SEM. GraphPad Prism 8.0 software (San Diego, CA, USA) was used for the statistical analysis. Western blotting and immunofluorescence analyses were performed using the Image J software. Data among multiple groups were compared using one-way analysis of variance, followed by a post hoc correction using Tukey’s test. a student’s t-test was used to test the difference between two groups. A value of *p < 0.05 was indicative of a statistically significant difference.

### Supplementary information

Supplementary material

Reporting-summary

## Data Availability

All data needed to evaluate the conclusions in the paper are present in the paper and/or the Supplementary Materials. Additional data related to this paper may be requested from the authors.
