# Supplementary material for: Engineered extracellular vesicle-encapsulated CHIP as novel nanotherapeutics for treatment of renal fibrosis
Source: NPJ Regen Med. 2024 Jan 13;9:3. doi: 10.1038/s41536-024-00348-0 (PMC10787844; doi:10.1038/s41536-024-00348-0)
Supplement: Supplementary file 1 — Supplementary material [file 41536_2024_348_MOESM1_ESM.pdf]

Supplementary material

Engineered extracellular vesicle-encapsulated CHIP as novel nanotherapeutics for treatment of renal fibrosis

Cheng Ji[1,2 #], Jiahui Zhang[2#], Linru Shi[2, #], Hui Shi[2], Wenrong Xu[2], Jianhua Jin[1*], Hui Qian[1,2*]

[1] Wujin Institute of Molecular Diagnostics and Precision Cancer Medicine of Jiangsu University, Wujin Hospital Affiliated with Jiangsu University, Chang Zhou, Jiangsu 213017, China

[2] Jiangsu Key Laboratory of Medical Science and Laboratory Medicine, School of Medicine, Department of laboratory Medicine, Jiangsu University, 212013 Zhenjiang, China

[*]Corresponding author. Hui Qian, E-mail: lstmmmlst@163.com; Jianhua Jin, E-mail: jianhuajin88@sina.com

[#]These authors contributed equally to this work.

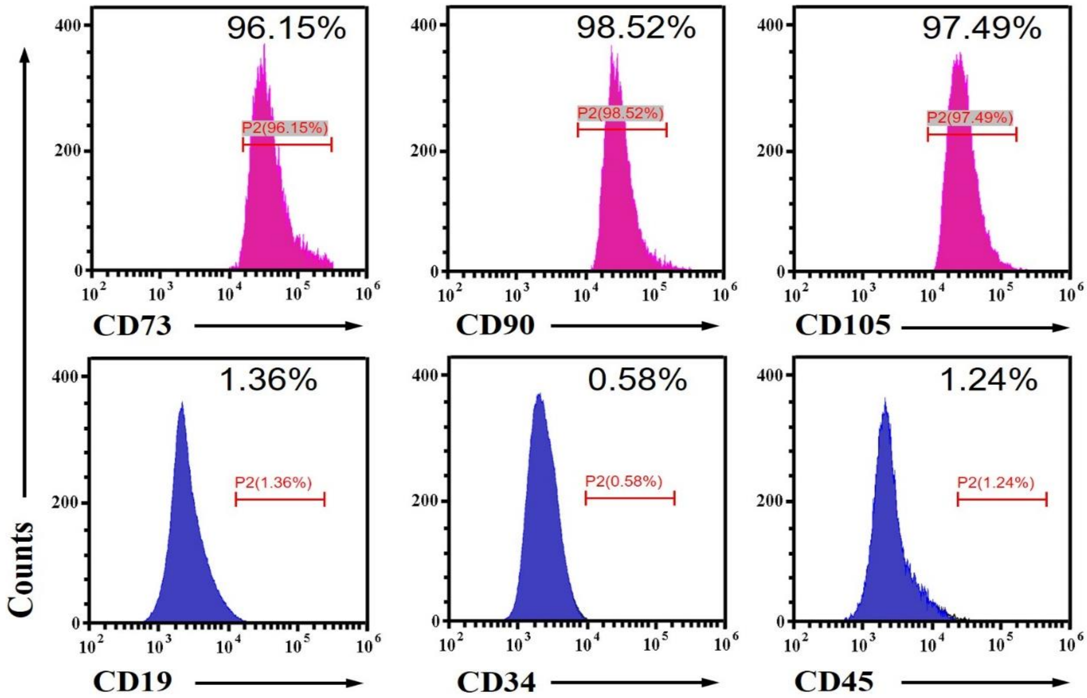

Supplementary Figure 1. Immunophenotypes of the human umbilical cord derived MSCs (hucMSCs) were measured by flow cytometry, positive expression of CD73, CD90, and CD105, negative expression of CD19, CD34, and CD45.

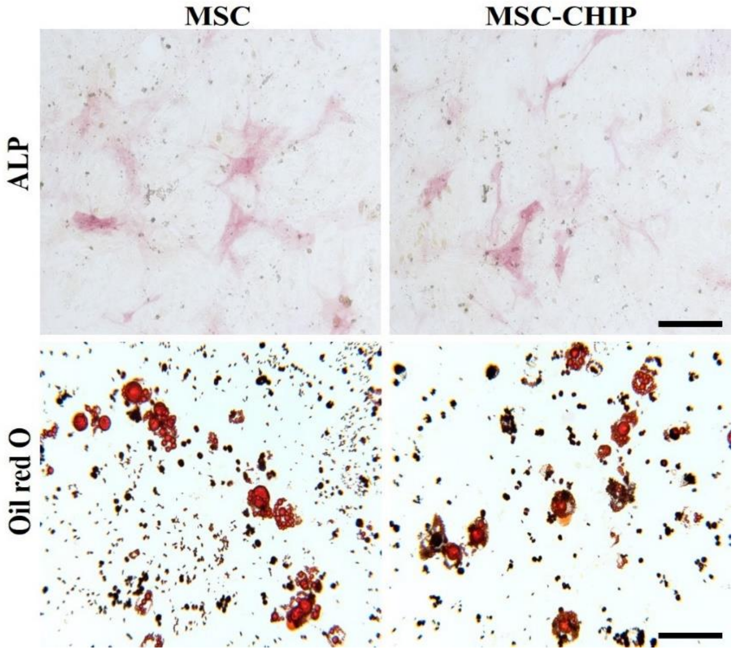

Supplementary Figure 2. Alkaline phosphatase (ALP) staining was used to detect the osteogenesis of MSC and MSC-CHIP group (above). Scale bar:100μm. Oil red O staining for detecting lipogenesis in MSC and MSC-CHIP group (below). Scale bar:500μm.

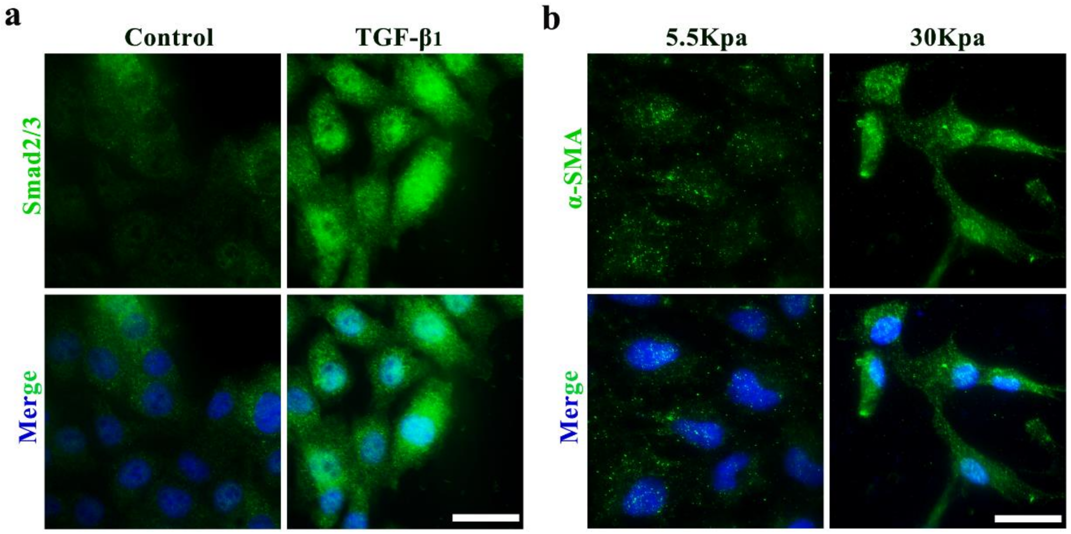

Supplementary Figure 3. (a) The renal tubular cells (NRK-52E cell line) were pretreated with 10nM TGF-β_1 protein for 24 h. The Smad2/3 (Green) was observed. Nuclei were stained with DAPI (Blue). Scale bar: 25 μm. **(b)** NRK-52E cells were stimulated with 5.5Kpa/30Kpa stiff gel for 24 h. Confocal observed the fibrosis index α-SMA (Red). Nuclei were stained with DAPI (Blue). Scale bar: 25 μm.

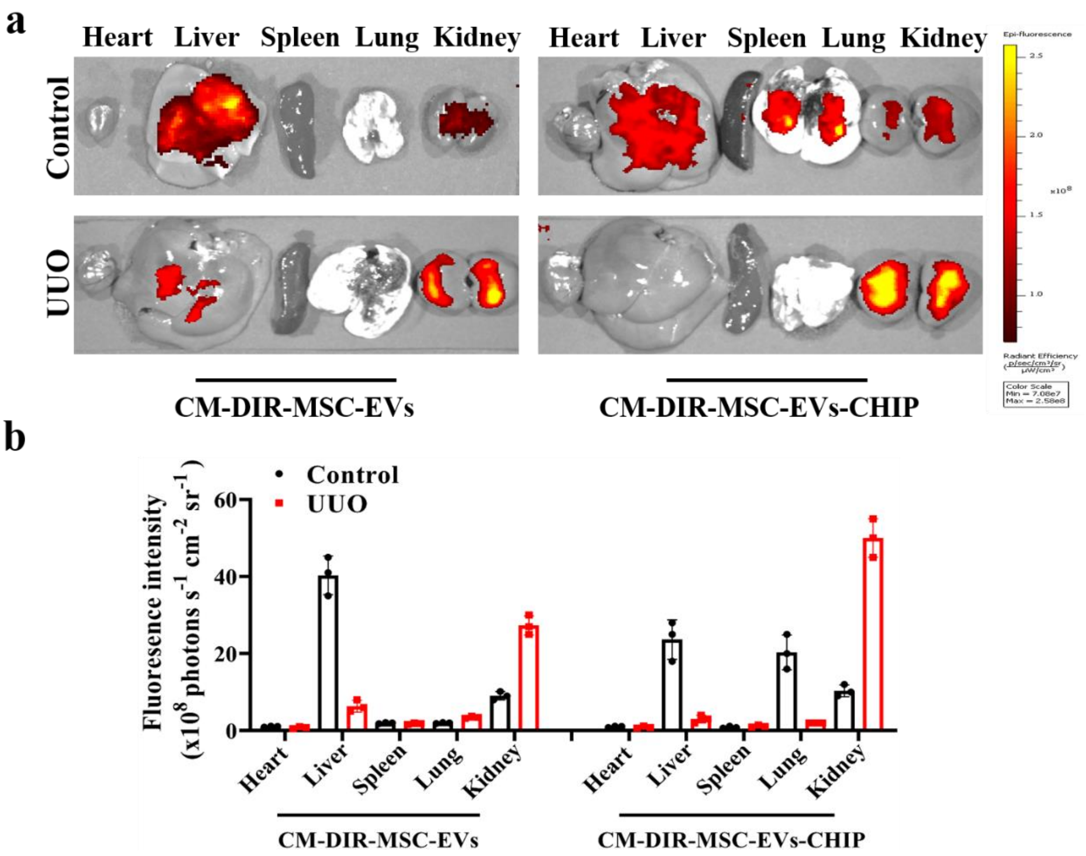

Supplementary Figure 4. (a) Intravenously injected with CM-DIR-labeled MSC-EVs and MSC-EVs-CHIP for 48 h in Control and UUO model. Ex vivo fluorescence images of CM-DIR-labeled EVs in major organs (n = 3). **(b)** Fluorescence intensity per gram of tissue in heart, liver, spleen, lung and kidney in Control and UUO model. (n = 3). Data are represented as the mean ± S.E.M. Statistical significance was calculated by a student's t test. ns, non-significance, **P* < 0.05, ***P* < 0.01, ****P* < 0.001.

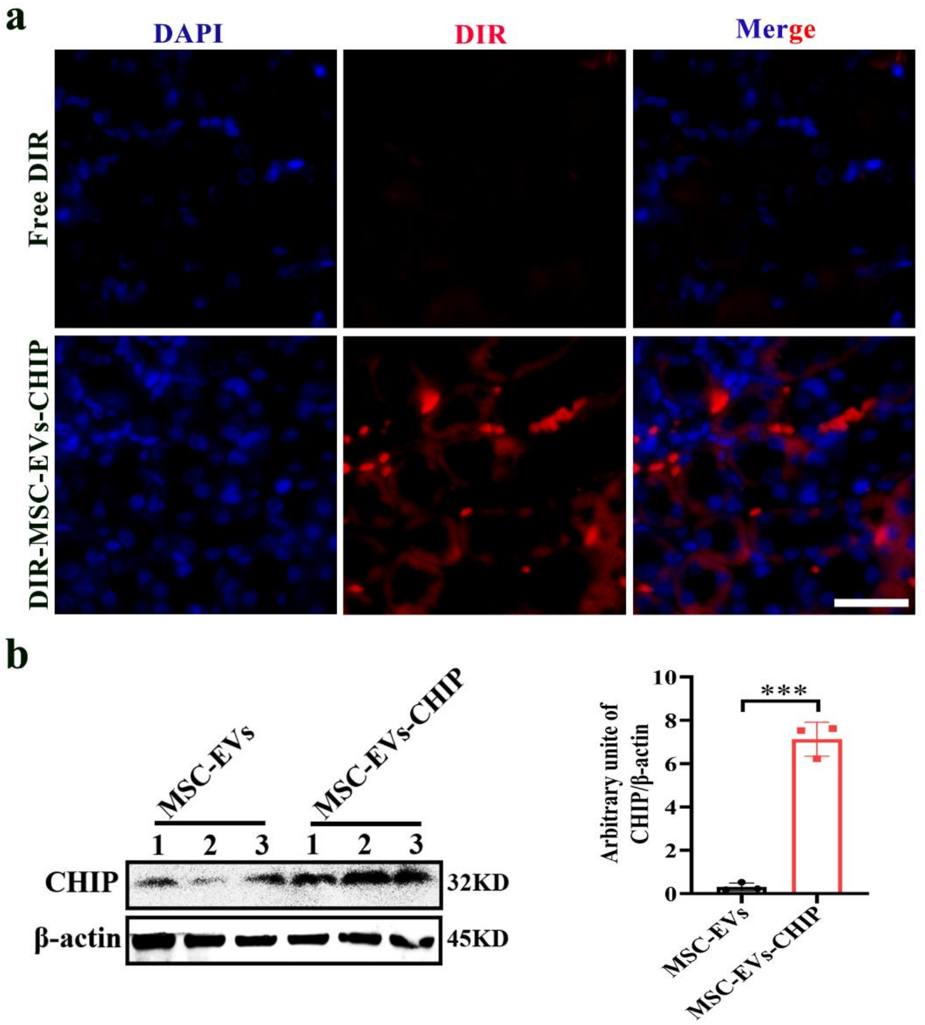

Supplementary Figure 5. (a) Representative images of DIR fluorescence in renal of UUO rat (n = 3). Nuclei were stained with DAPI (Blue). Scale bar: 100 μm. **(b)** The expression of CHIP in renal of UUO rat was determined by western blotting (n = 3) (left). The relative quantification of CHIP (n = 3) (right). Data are represented as the mean ± S.E.M. Statistical significance was calculated by a student's t test. ns, non-significance, *$P < 0.05$, **$P < 0.01$, ***$P < 0.001$.

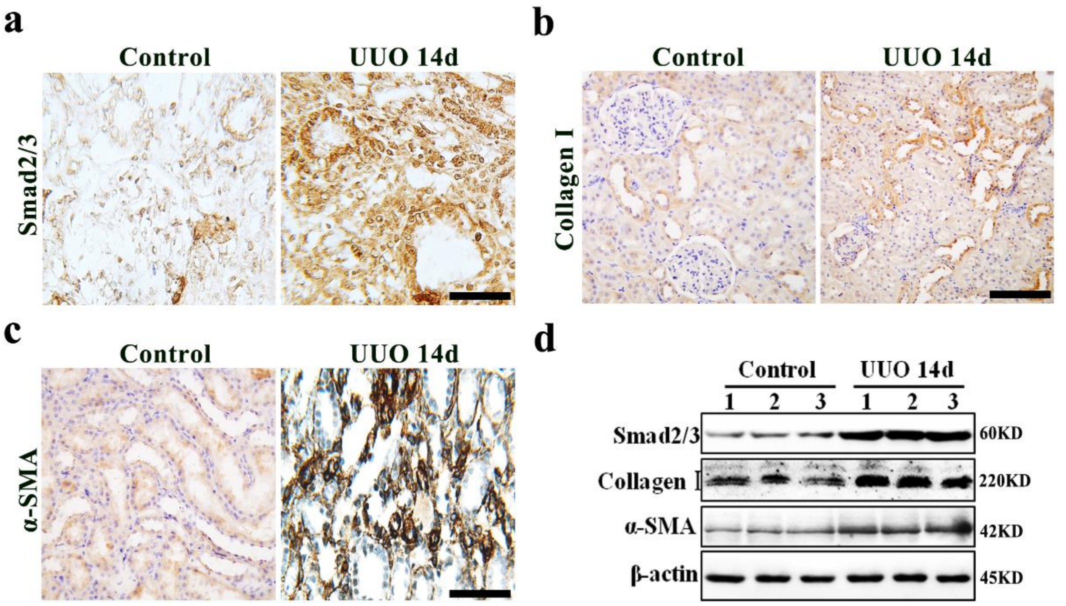

Supplementary Figure 6. (a) Representative images of Smad2/3 in UUO rat were demonstrated by immunohistochemistry (n = 3). Scale bar: 100 μm. **(b)** Representative images of Collagen I **c)** α-SMA were demonstrated by immunohistochemistry. Scale bar: 100 μm. **(d)** The expression of Smad2/3, Collagen I and α-SMA in UUO renal tissue was determined by western blotting

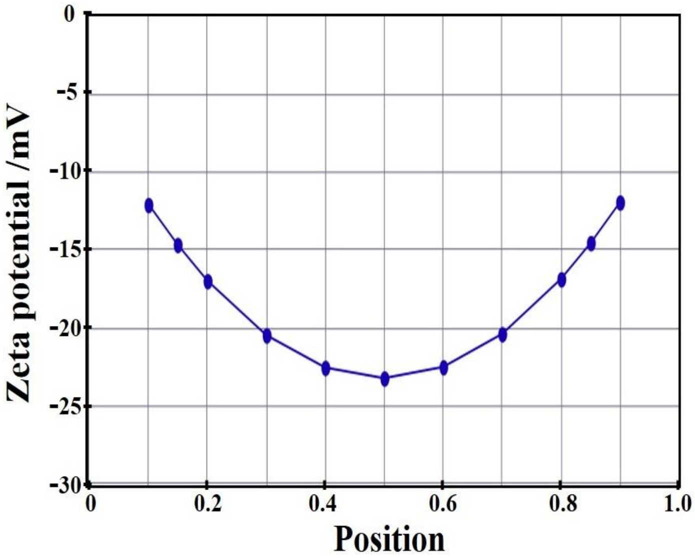

Supplementary Figure 7. The zeta potential of SPION-EVs.

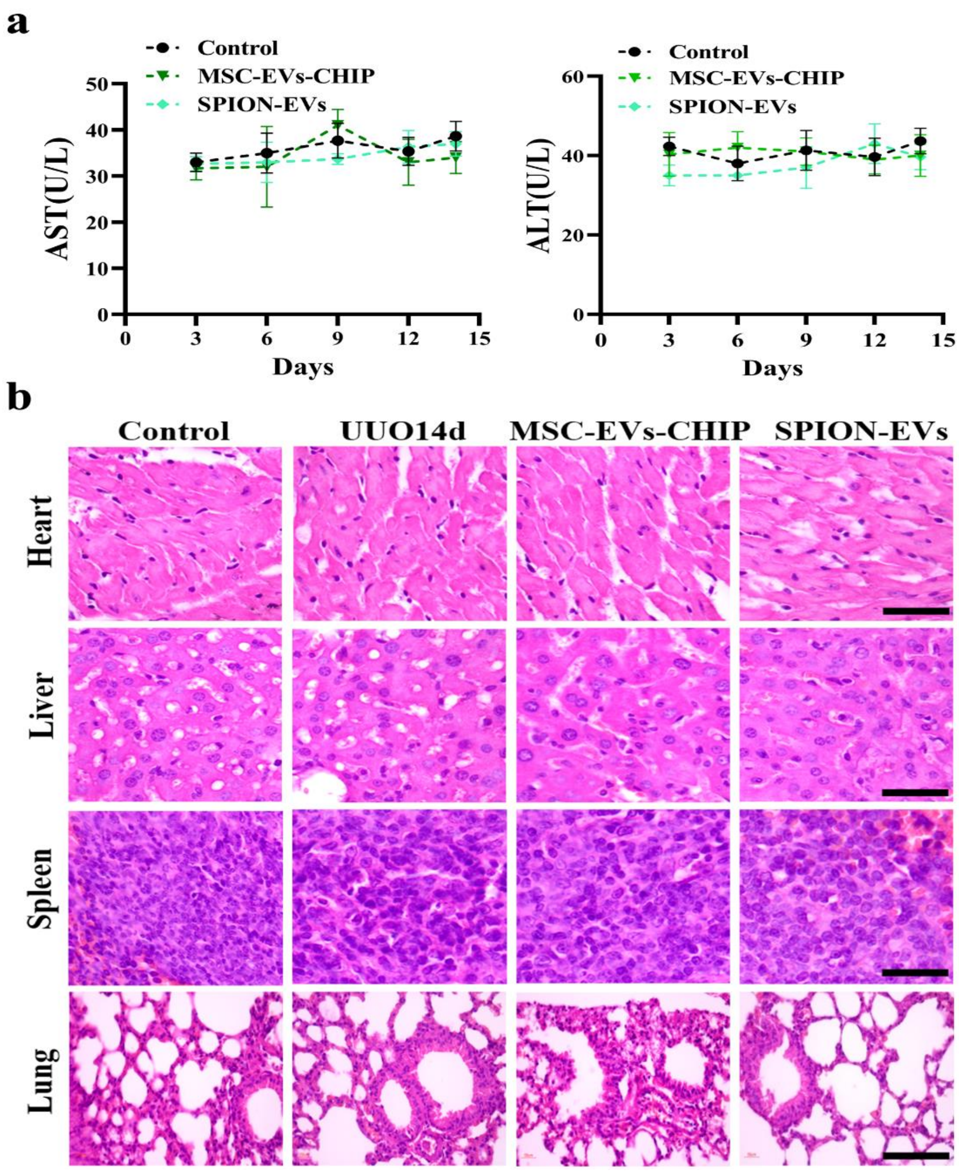

Supplementary Figure 8. (a) Blood biochemical tests for rat liver function indicators in different groups. **(b)** Histological analyses of major organs in different groups. Scale bar: 100 μm.

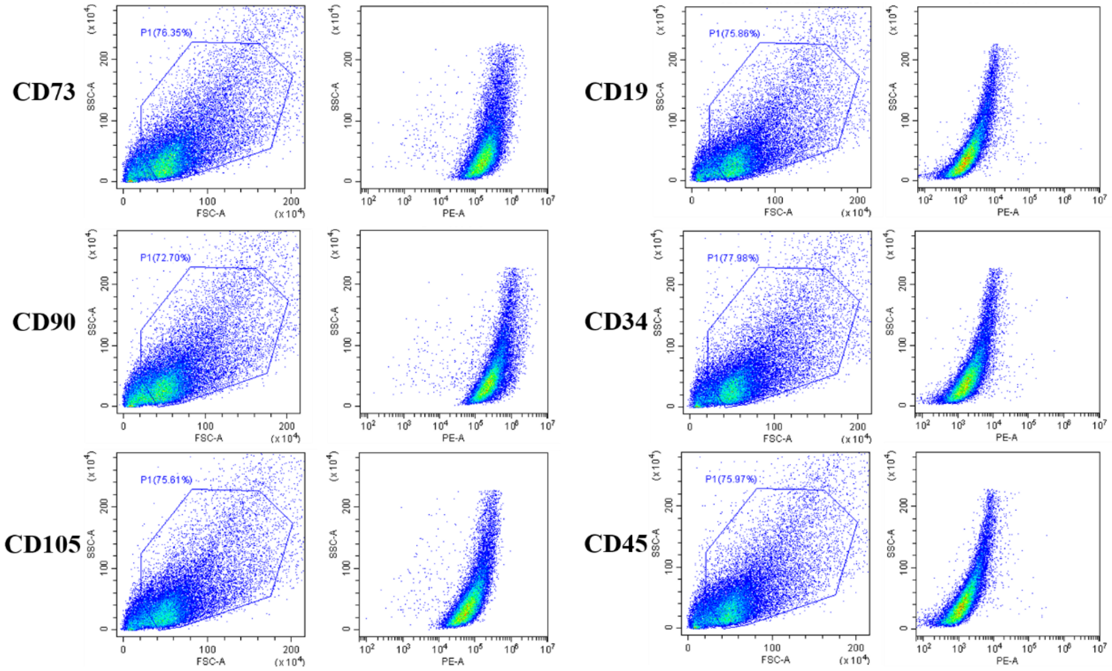

Supplementary Figure 9. FACS sequential gating strategy of Supplementary Figure 1. Total human umbilical cord derived MSCs were first gated on a forward scatter (FS)/side scatter (SS) plot to get the single cell, then gated on the CD73$^+$, CD90$^+$, and CD105$^+$ population, and gated on the CD19$^+$, CD34$^+$, and CD45$^+$ population.

Unedited images for Fig. 1g

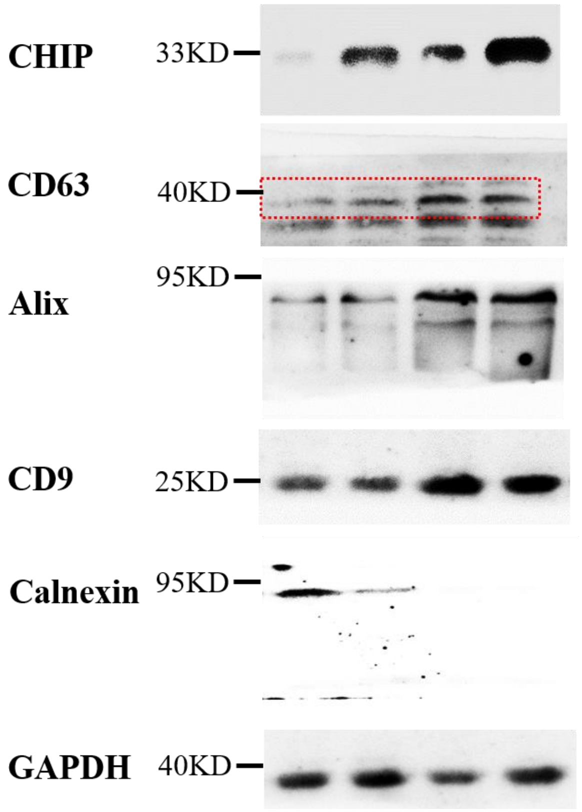

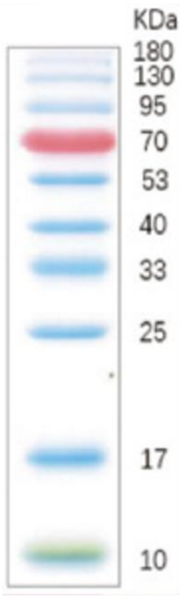

Tris-Glycine 15%

Marker (Sigma-Aldrich, 94964)

Unedited images for Fig. 2c

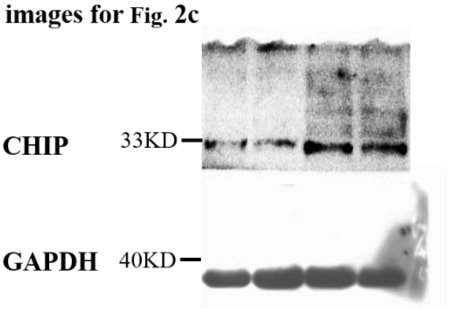

Unedited images for Fig. 2e

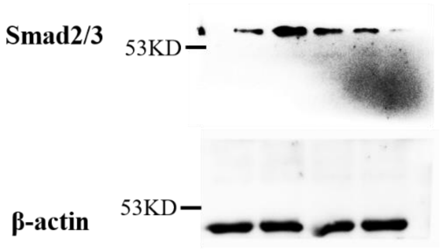

Unedited images for Fig. 2g

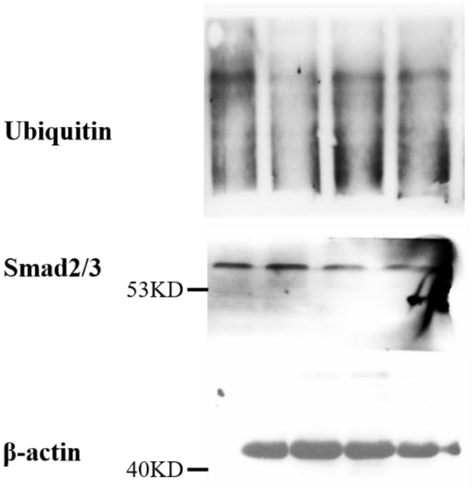

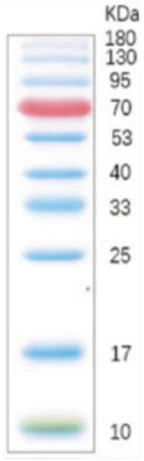

Tris-Glycine 15%
Marker (Sigma-Aldrich, 94964)

Unedited images for Fig. 3d

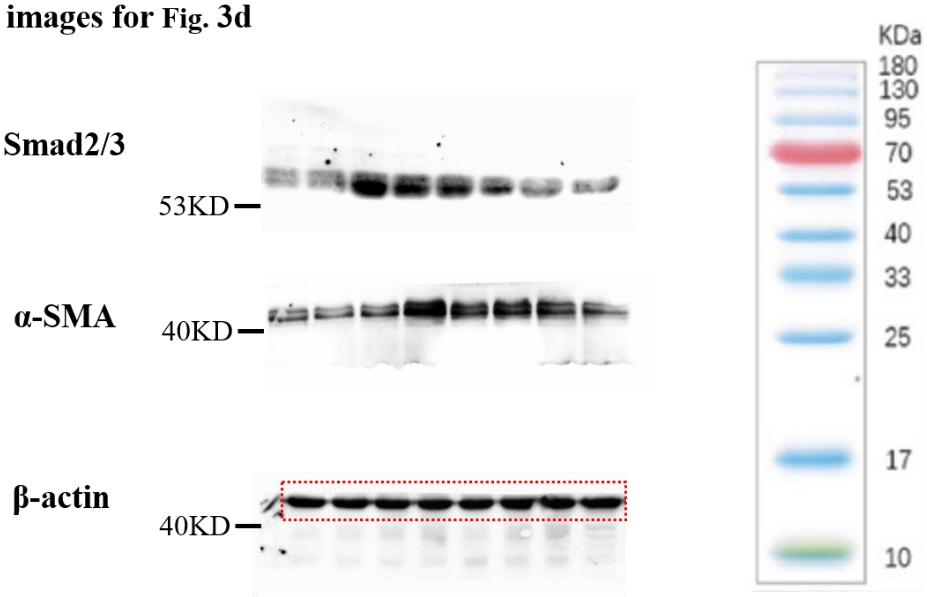

Tris-Glycine 15%
Marker (Sigma-Aldrich, 94964)

Unedited images for Fig. 4i

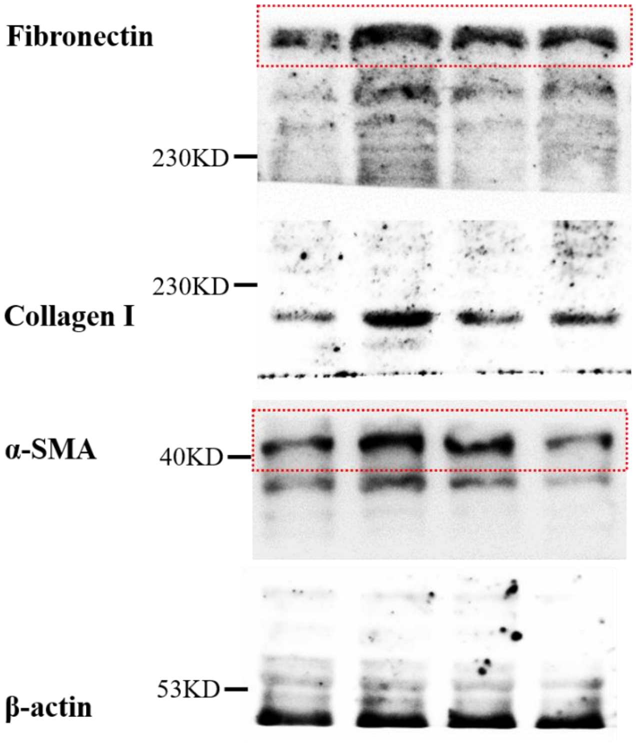

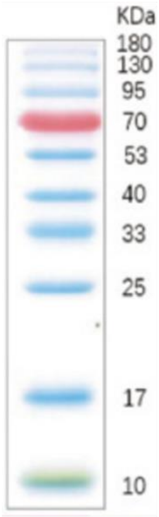

Tris-Glycine 15%

Marker (Sigma-Aldrich, 94964)

Unedited images for Fig. 5g

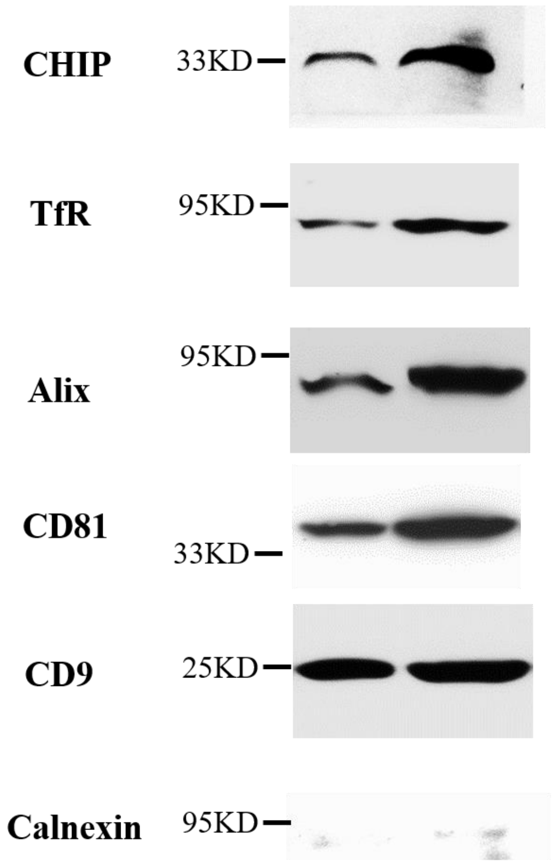

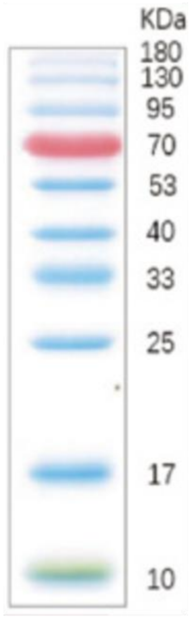

Tris-Glycine 15%

Marker (Sigma-Aldrich, 94964)

Unedited images for Fig. 6g

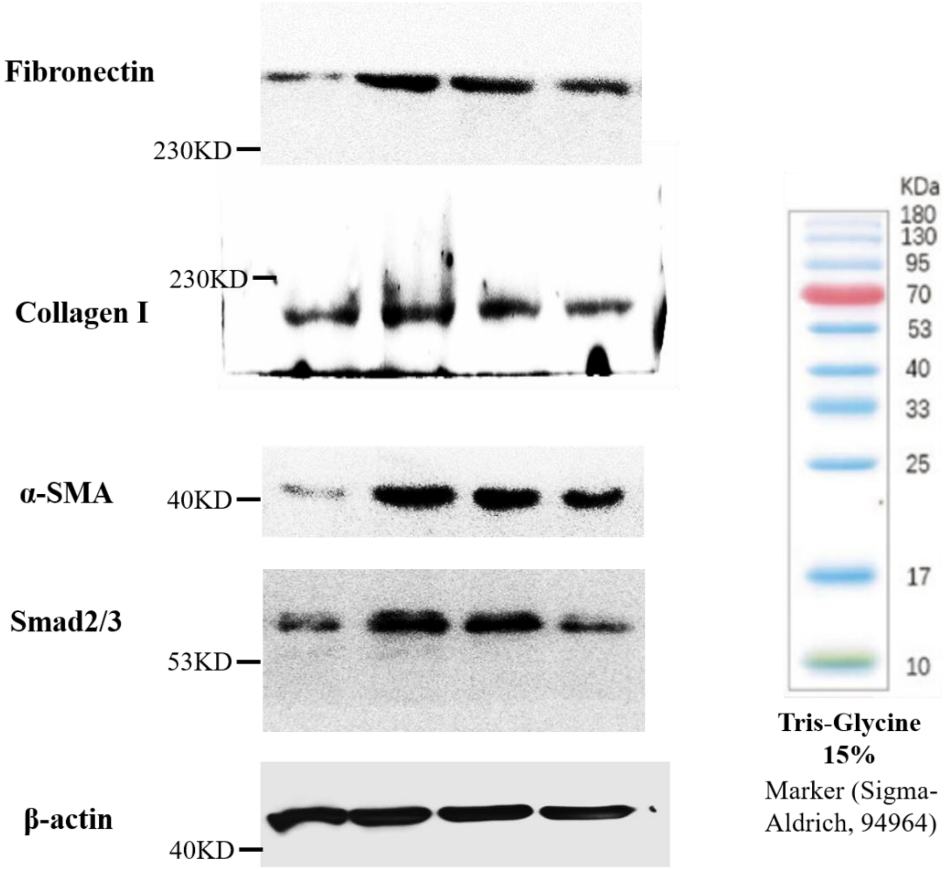

Tris-Glycine 15%

Marker (Sigma-Aldrich, 94964)

Unedited images for Supplementary Fig. 5b

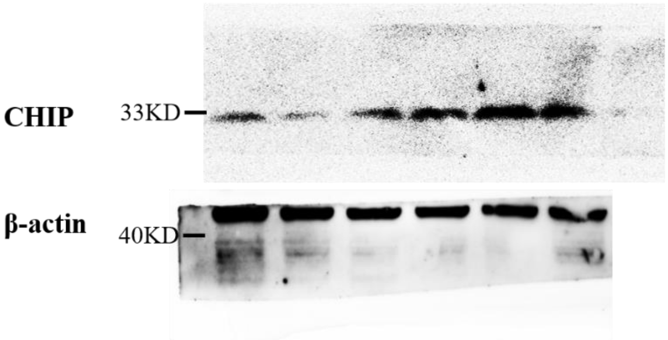

Unedited images for Supplementary Fig. 6d

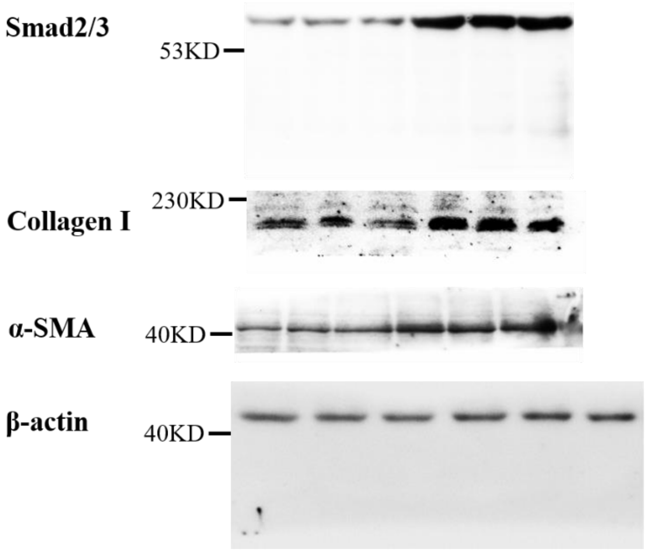

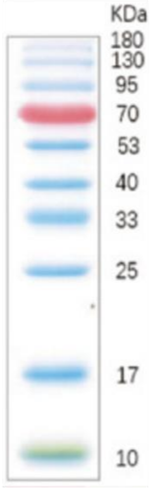

Tris-Glycine 15%

Marker (Sigma-Aldrich, 94964)

Supplementary Figure 10. The uncropped and unprocessed images of western blots shown in the manuscript.
